# Treatment of patients with spinal cavernous malformations: a systematic review

**DOI:** 10.1016/j.bas.2026.106021

**Published:** 2026-03-25

**Authors:** Abel Clemens Adriaan Sandmann, Marinus Abraham Kempeneers, K. Mariam Slot, René van den Berg, William Peter Vandertop, Dagmar Verbaan, Jonathan M. Coutinho

**Affiliations:** a Amsterdam UMC, University of Amsterdam, Department of Neurology, Amsterdam, the Netherlands; b Amsterdam Neuroscience, Neurovascular Disorders, Amsterdam, the Netherlands; c Amsterdam UMC, University of Amsterdam, Department of Neurosurgery, Amsterdam, the Netherlands; d Amsterdam UMC, University of Amsterdam, Department of Radiology and Nuclear Medicine, Amsterdam, the Netherlands

**Keywords:** Cavernous malformation, Spinal cord, Intramedullary, Treatment, Surgery, Conservative management

## Abstract

**Introduction:**

Spinal cavernous malformations (SCM) are intramedullary vascular malformations that can be treated surgically or conservatively.

**Research question:**

What is the optimal treatment strategy for patients with SCM?

**Material and methods:**

We searched Medline, Embase, Web of Science, and Cochrane Library (until 3 April 2025) for studies evaluating treatment outcomes of ≥5 patients with SCM of all ages. Two independent reviewers assessed study eligibility and risk-of-bias using ROBINS-I. We descriptively analyzed change in neurological outcome, independent ambulation, and hemorrhage rates.

**Results:**

Of 1293 articles screened, we included 50 studies reporting on 2328 patients with SCM (mean age 42 years [SD ± 14], 47% women). Studies were predominantly retrospective (48 [96%]) and single-center (46 [92%]). Overall risk-of-bias was serious in 80% and moderate in 20% of studies. The pooled annual hemorrhage rate during 649 person-years of follow-up was 4.8% (95% CI 3.2%-6.8%). After surgical treatment (n = 1909 [82%], mean follow-up 43 months [SD ± 38]), the neurological outcome improved in 45%, was stable in 41%, and worsened in 9%, and 72% ambulated independently versus 60% preoperatively. After conservative management (n = 419 [18%], mean follow-up 47 months [SD ± 34]), the neurological outcome improved in 16%, was stable in 69%, and worsened in 10%, and 76% ambulated independently versus 79% at baseline.

**Discussion and conclusion:**

The majority of patients with SCM were treated surgically and had worse baseline outcomes than those managed conservatively. Approximately three-quarters ambulated independently after either strategy. Low methodological quality and high risk-of-bias complicated direct comparisons, precluding definitive conclusions. Prospective, multicenter studies are warranted.

## Highlights

• The annual hemorrhage risk from spinal cavernous malformations is approximately 5%.
• The surgery group was larger and more severely affected than the conservative group.
• Neurological outcome improved more often with surgery than conservative management.
• Around three-quarters of patients ambulated independently after either strategy.
• Prospective, multicenter studies are needed to better inform treatment decisions.

## 1 Introduction

Spinal cavernous malformations (SCM) are intradural, intramedullary vascular malformations that may also have an extramedullary component (Cosgrove et al., 1988). SCMs are rare, accounting for approximately 5% of structural spinal lesions (Da Ros et al., 2021; Choi et al., 2011), and around 5% of all cavernous malformations in the central nervous system (Goldstein and Solomon, 2017). Due to widespread availability of MRI, SCMs are increasingly detected and the number of cases reported in the literature has increased substantially over the last decades (Santoro et al., 2004; Velz et al., 2018). Unlike cerebral cavernous malformations (CCM), SCMs may more readily cause neurological symptoms due to the eloquence of the spinal cord and its limited ability to compensate for space-occupying lesions or episodes of hemorrhage (Sandmann et al., 2024; Maslehaty et al., 2011).

Clinical decision-making for patients with SCM is complicated by a scarcity of large studies. Many single-center case series have recommended surgical treatment, advocating for early intervention despite potential risks of complications (Ogilvy et al., 1992; Spetzger et al., 1995; Tu et al., 1999). However, these studies often lacked a control group, precluding conclusions of a potential benefit over conservative management. In recent literature, patients with SCM who were managed conservatively are increasingly reported (Ren et al., 2022; Santos et al., 2022), with several studies reporting favorable results (Kharkar et al., 2007). Nevertheless, studies that directly compare surgical treatment and conservative management remain sparse.

In current clinical practice, the management of patients with SCM is informed by previously published literature syntheses (Asimakidou et al., 2022; Badhiwala et al., 2014; Fotakopoulos et al., 2021; Rios-Zermeno et al., 2025). These reviews are, however, limited by their absence of baseline characteristics stratified by treatment strategy (Asimakidou et al., 2022; Badhiwala et al., 2014), use of quantitative meta-analysis despite substantial heterogeneity and confounding factors in the included studies (Badhiwala et al., 2014; Fotakopoulos et al., 2021; Rios-Zermeno et al., 2025), calculation of pooled annual hemorrhage rates from birth (Badhiwala et al., 2014), and small sample size (Rios-Zermeno et al., 2025). Therefore, our objectives were to systematically search and review all available literature on the treatment of SCM, conduct a descriptive analysis of surgical and conservative treatment groups with attention to sources of bias, and provide recommendations for future research.

## 2 Materials and methods

This systematic review was conducted in accordance with the Preferred Reporting Items for Systematic reviews and Meta-Analyses (PRISMA) guidelines (Page et al., 2021). A study protocol outlining the objectives, search strategy, eligibility criteria, study outcomes, and data extraction and synthesis methods was developed in accordance with the PRISMA-P guidelines (Shamseer et al., 2015), and was registered in PROSPERO register before data extraction was started (CRD42024572222). The majority of data supporting the findings of this review are included in the Supplemental File. Additional data may be shared upon reasonable request to the corresponding author. Institutional review board approval and patient consents were not applicable to this review of only previously published literature.

### 2.1 Search strategy

We performed a comprehensive literature search in Medline (PubMed), Embase, Web of Science, and Cochrane Library databases without restrictions for publication date or language for studies published up to 3 April 2025. The search strategy included relevant terms for ‘SCM’, ‘surgical treatment’, and ‘conservative management’ (Supplemental Table 1). Furthermore, we manually searched the reference lists of the included articles to identify additional relevant studies.

### 2.2 Study selection

Two reviewers (ACAS and MAK) independently screened all search results by title and abstract, and assessed full texts of potentially eligible articles. Any disagreements were resolved through discussion. Studies were eligible for inclusion if they reported on patients with SCM of all ages who underwent surgical treatment, conservative management, or both, evaluating at least one outcome of interest over a specified follow-up period. Exclusion criteria were: (1) other disease than cavernous malformation; (2) other locations than intradural; (3) less than five patients; (4) unknown outcomes or follow-up (i.e. period of monitoring); (5) in vitro or animal studies; (6) incorrect article type (review, meta-analysis, case report, conference abstract, letter, or book chapter); or (7) full-text article not available.

Study authors were contacted to request full texts when these were not available. Studies published in languages other than English were included if an English-translated abstract was available. The full text of these articles was subsequently translated. If multiple publications reported on partially overlapping patients from the same cohort, we included the largest, most complete study to prevent double reporting of cases and recorded the studies excluded for this reason. Publications based on the exact same patient cohort but reporting different outcomes were included.

### 2.3 Data extraction

Data were extracted from the included studies using a structured record form. These data included study characteristics, patient and lesion characteristics, details on treatment and follow-up, and outcomes of interest. Study characteristics comprised the country and city where the study was conducted, as well as the study period and design. Data on patient characteristics included the number of patients, age (mean and standard deviation [SD]), sex, and mode of presentation (i.e. symptomatic or asymptomatic). Among symptomatic patients, we reported the type of symptoms (e.g. motor or sensory), and the type of clinical presentation according to Ogilvy et al. (1992), modified Ogilvy (Ren et al., 2019), or Choi et al. (2011), if available. Data on lesion characteristics included the location of SCM at the spinal cord and in the horizontal plane (i.e. superficial or deep). SCMs were classified as superficial if they were in contact with the pial surface either on MRI or intraoperatively (Zhang et al., 2016; Ohnishi et al., 2020). Lastly, we recorded the largest diameter of SCM (mean and SD). If possible, these baseline characteristics were stratified by treatment strategy. Studies with missing data were not excluded, but we reported the number of patients and studies available for each characteristic.

Treatment details included whether surgical treatment and/or conservative management was performed. For surgically treated patients, we recorded the duration of symptoms, depicting the time from symptom onset to the surgical procedure, unless otherwise specified. Additionally, we noted whether during the procedure intraoperative neuromonitoring (IONM) was used and the extent of surgical resection (i.e. complete or incomplete). The follow-up duration until final follow-up started at surgical intervention for surgically treated patients and at diagnosis for those managed conservatively.

### 2.4 Outcomes of interest

We evaluated change in neurological outcome, categorized as improved, stable, or worsened, as reported in the included articles, regardless of how neurological change was defined. In patients who were treated surgically, change from the preoperative status was measured postoperatively and at final follow-up. The postoperative assessment was conducted immediately after surgery (Wachter et al., 2012; Reitz et al., 2015), at discharge (Velz et al., 2018), or by the end of the first or second postoperative week (Spetzger et al., 1995; Li et al., 2018). In patients who were managed conservatively, change from baseline was only measured at final follow-up. We further calculated pooled annual hemorrhage rates. Hemorrhage rates of individual studies were extracted as defined in the articles, regardless of potential variations in the definition of hemorrhage from SCM. Additionally, for patients treated with surgery, we assessed the occurrence of postoperative complications and (re)hemorrhages, which studies reported separately from neurological worsening in most cases. Studies were not excluded if they did not report each outcome of interest or had missing data. Instead, we provided the number of patients and studies available for each outcome.

Additional outcomes were assessed using several standardized scoring tools: McCormick (McCormick et al., 1990), modified McCormick (Choi et al., 2011), and Frankel (Frankel et al., 1969) scales, as well as the Aminoff-Logue Disability Scale (ALDS) (Aminoff and Logue, 1974) and the American Spinal Injury Association (ASIA) Impairment Scale (AIS) (Maynard et al., 1997). Details of these outcome tools are provided in Supplemental Table 2. To facilitate comparisons across studies using different tools, standardized outcome tools used in surgically and conservatively treated patients were transformed into a dichotomous outcome whether patients could ambulate independently without the need for external aid or not. McCormick 1-2, modified McCormick 1-2, and Frankel D-E scores reflected independent ambulation, while McCormick 3-4, modified McCormick 3-5, and Frankel A-C scores represented an inability to ambulate independently. If studies used multiple different outcome tools in the same patients, we included only one tool, prioritizing the (modified) McCormick scale over the Frankel scale.

### 2.5 Quality assessment

Two reviewers (ACAS and MAK) independently assessed the methodological quality of the included studies using the Risk Of Bias In Non-randomized Studies of Interventions (ROBINS-I) tool (Sterne et al., 2016). Any discrepancies were discussed until consensus was reached. The ROBINS-I tool contains seven domains of bias, consisting of several signaling questions, leading up to a risk-of-bias judgment in that domain of low, moderate, serious, critical, or no information. Based on the domain-level judgments, an overall judgment is made. At least one domain-level judgment of moderate, serious, or critical risk of bias leads to an overall risk judgment of moderate, serious, or critical, respectively. An overall critical risk of bias precludes a study from further synthesis in meta-analysis, but since the current review does not involve meta-analysis, we only used low, moderate, and serious ratings. Results of the risk-of-bias assessment were visualized using the ROBVIS tool (McGuinness and Higgins, 2020).

### 2.6 Statistical analysis

We conducted a descriptive analysis, evaluating outcomes across the included studies while considering the comparability of treatment groups at baseline. We did not perform meta-analysis due to anticipated heterogeneity between studies, variability in the measurement of outcomes, and risk of bias due to the lack of a control group in many studies. Due to the absence of meta-analysis, we did not formally assess the certainty of synthesized outcomes. We qualitatively assessed whether reporting bias could be present by noting absence of expected outcomes in the included studies. In addition to the overall analysis of all included studies reporting on patients who underwent surgical treatment, conservative management, or both, we conducted a comparative cohort analysis using only those studies that reported on both surgical treatment and conservative management, in order to address the limitation of studies evaluating only a single treatment strategy.

We summarized each numerical variable by calculating weighted means according to the sample size (excluding studies that only provided a median value), and pooled SDs by combining the variances from each study weighted by their sample size (excluding studies that only reported ranges or interquartile ranges [IQR]). Counts were summarized by summation and calculation of proportions. To evaluate the comparability of treatment outcomes, we tested the characteristics of patients in each treatment group at baseline, using the unpaired *t*-test for continuous data (taking into account the weighted mean and pooled SD) and the Chi-squared test for categorical data (or the Fisher's exact test if there was an expected cell count less than five). Syntheses of the change in neurological outcome and proportions of patients who ambulated independently were visualized using proportional stacked bars.

Pooled annual hemorrhage rates were calculated after presentation during follow-up without or before surgical intervention, using the sum of follow-up in person-years. We performed a per-patient analysis, including only the earliest hemorrhage observed during follow-up if follow-up was subsequently censored. In studies that did not censor follow-up after the first bleed, we calculated a per-hemorrhage (or incidence) rate, including the total number of hemorrhages divided by the overall (uncensored) follow-up. In studies that assumed SCMs were congenital, annual hemorrhage rates were calculated from birth. These rates were pooled (by dividing the total number of hemorrhages by the sum of person-age-years from birth to presentation or birth to surgery) but not presented in the main text. Analyses were done using IBM SPSS Statistics version 28 (IBM Corp. Released, 2021).

## 3 Results

### 3.1 Study selection and characteristics

**Fig. 1:**
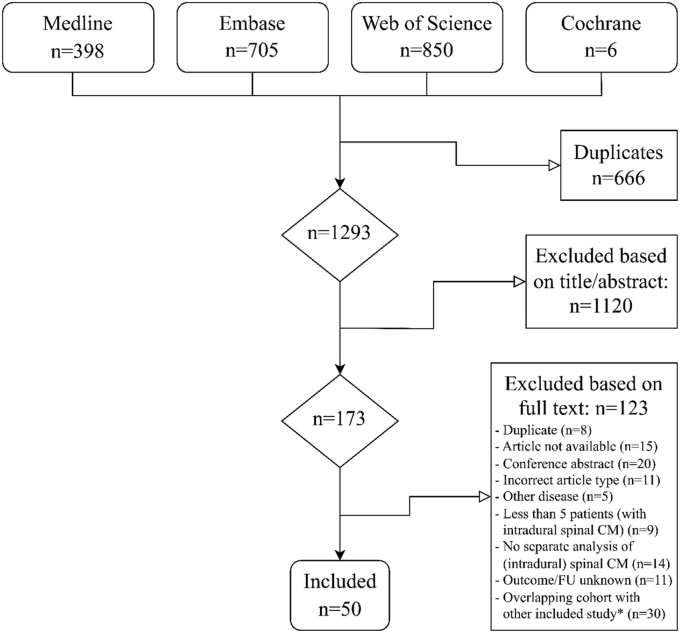
Flow diagram of study selection; ∗Thirty articles were excluded because the cohorts partially overlapped with an included larger, more complete study (Supplemental Table 3); CM, cavernous malformation; FU, follow-up.

The search yielded 1959 studies. After removing duplicates, 1293 articles were screened by title and abstract (Fig. 1). Following the full-text assessment of 173 articles, 50 studies reporting on 5 to 279 patients were included. Thirty publications were excluded because the cohorts partially overlapped with a larger, more complete study that was included (Supplemental Table 3). We did not identify separate publications reporting different outcomes of the exact same patient cohort.

Characteristics of the included studies are summarized in Table 1 (with additional study-level details provided in Supplemental Table 4). Studies were published between 1988 and 2025, with 34 (68%) studies published in or after 2010. All studies were conducted in the Northern Hemisphere (11 in North America, 15 in Europe, and 24 in Asia, Supplemental Fig. 1). The majority of studies were retrospective (48 [96%]) and single-center (46 [92%]).

**Table 1:** Characteristics of the fifty included studies, their patients and the measured outcomes.

Authors and year	Country	SD	N	Age	F (%)	Sur	Con	Outcome(s) of interest
Cosgrove et al., 1988	Canada	RS	5	41	3 (60)	5	0	Noff
McCormick et al., 1988	USA	RS	6	33	2 (33)	6	0	Noff
Ogilvy et al., 1992	USA	RS	6	42	3 (50)	6	0	Noff
Cantore et al., 1995	Italy	RS	6	54	1 (17)	6	0	Noff
Spetzger et al., 1995	Germany	RS	9	43	3 (33)	9	0	Nopo, noff
Padovani et al., 1997	Italy	RS	6	45	2 (33)	6	0	Noff
Tu et al., 1999	Taiwan	RS	7	30	2 (29)	7	0	Nopo, noff
Shan et al., 2002	China	RS	23	32	10 (43)	21	2	Nopo, noff
Sandalcioglu et al., 2003	Germany	RS	10	38	7 (70)	10	0	Nopo, noff, Frankel
Santoro et al., 2004	Italy	RS	10	41	5 (50)	10	0	Nopo, noff
Jallo et al., 2006	USA	RS	26	38	9 (35)	26	0	Nopo, noff
Kharkar et al., 2007	USA	RS	14	42	6 (43)	4	10	Noff, mMS
Gu et al., 2008	China	RS	28	36	16 (57)	28	0	Noff
Labauge et al., 2008	France	RM	53	40	27 (51)	40	13	Noff, MS
Bian et al., 2009	China	RS	16	38	9 (56)	16	0	Noff, Frankel
Park et al., 2009	Korea	RS	14	34	5 (36)	14	0	Nopo, noff
Deutsch 2010	USA	RS	5	56	2 (40)	5	0	Noff, HR
Steiger et al., 2010	Germany	RS	20	39	11 (55)	17	3	Noff, Frankel
Aoyama et al., 2011	Japan	RS	12	33	5 (42)	12	0	Noff, MS
Choi et al., 2011	Korea	RS	21	39	13 (62)	21	0	Nopo, noff, mMS
Maslehaty et al., 2011	Germany	RS	11	42	9 (82)	11	0	Nopo, noff, MS, Frankel
Hegde et al., 2012	Singapore	RS	6	39	3 (50)	0	6	Noff
Wachter et al., 2012	Germany	RS	30	42^a^	17 (57)	30	0	Nopo, noff
Kim et al., 2013	Korea	RS	24	52	11 (46)	0	24	HR
Badhiwala et al., 2014	Canada	RS	24	40	10 (42)	11	13	Nopo, noff
Li et al., 2014	China	RS	21	39	8 (38)	21	0	Noff
Reitz et al., 2015	Germany	RS	48	41	23 (48)	48	0	Nopo, noff, AIS
Zhang et al., 2016	China	RS	85	41	34 (40)	58	27	Noff, mMS, HR
Imagama et al., 2017	Japan	PS	41	39	23 (56)	41	0	mMS
Sun et al., 2017	Türkiye	RS	10	41	5 (50)	10	0	Noff, MS
Azad et al., 2018	USA	RS	32	44	19 (59)	32	0	Noff, Frankel, ALDS
Ghobrial et al., 2018	USA	RS	13	51	?	13	0	mMS
Li et al., 2018	China	RS	83	39	43 (52)	83	0	Nopo, noff, MS
Velz et al., 2018	Switzerland	RS	29	45	16 (55)	21	8	Nopo, noff, Frankel
Goyal et al., 2019	USA	RS	85	48	40 (47)	21	64	Noff, HR
Nagoshi et al., 2019	Japan	RS	66	45	30 (45)	57	9	mMS, AIS
Ohnishi et al., 2020	Japan	RS	18	37	7 (39)	5	13	mMS
Zhang et al., 2021	China	RS	111	41	51 (46)	111	0	Nopo, noff, mMS, ALDS
Zhang et al., 2021	China	RS	18	13	6 (33)	16	2	Nopo, noff, mMS
Liao et al., 2022	China	RS	98	42	35 (36)	98	0	Noff, AIS
Niedermeyer et al., 2022	Germany	RS	17	41	9 (53)	17	0	Noff, MS
Ren et al., 2022	China	RM	126	35	55 (44)	0	126	Noff, mMS, HR
Santos et al., 2022	Germany	RS	71	44	31 (44)	0	71	HR
Cai et al., 2023	China	RS	29	45	12 (41)	29	0	Noff, mMS
Chen et al., 2023	China	RS	30	48	16 (53)	19	11	Noff
Kurokawa et al., 2023	Japan	RS	160	52	70 (44)	160	0	Noff, mMS
Liu et al., 2023	China	PM	268	48	141 (53)	268	0	Noff, AIS
Srinivasan et al., 2023	USA	RS	146	45	77 (53)	146	0	Nopo, noff
Tian et al., 2023	China	RM	279	37	125 (45)	279	0	Noff, mMS
Früh et al., 2025	Germany	RS	52	54^a^	30 (58)	35	17	MS
**Synthesis**	**-**	**-**	**2328**	**42^b^**	**47%**	**1909**	**419**	**-**

Data are number, mean, number (%), or otherwise specified; all studies are cited in Supplemental Table 4; AIS, ASIA Impairment Scale; ALDS, Aminoff-Logue Disability Scale; ASIA, American Spinal Injury Association; Con, conservative management; F, female; HR, hemorrhage rate; mMS, modified McCormick Scale; MS, McCormick Scale; N, number; noff, neurological outcome at final follow-up; nopo, neurological outcome postoperatively; PM, prospective multicenter; PS, prospective single-center; RM, retrospective multicenter; RS, retrospective single-center; Sur, surgical treatment; SD, study design.

^1^Median.

^2^Weighted mean.

### 3.2 Risk-of-bias assessment

**Fig. 2:**
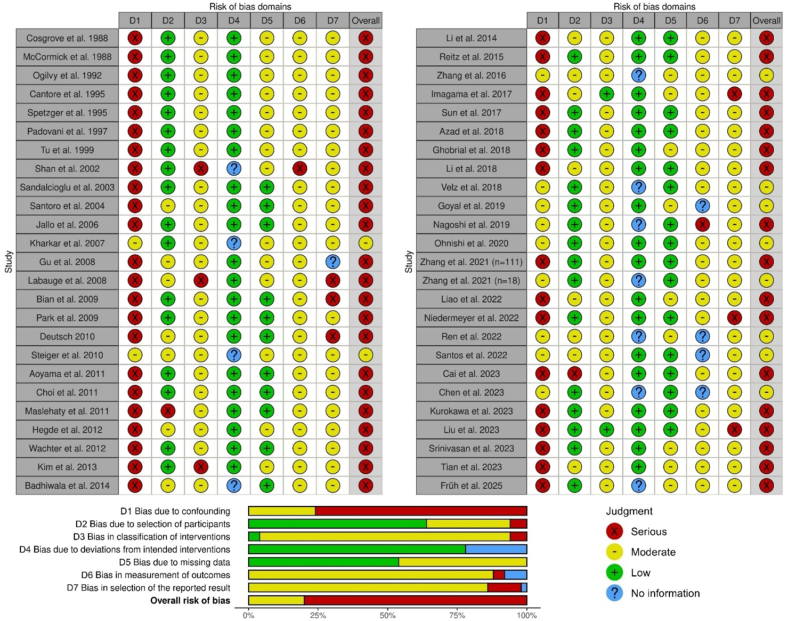
ROBVIS traffic light plot (McGuinness and Higgins, 2020) for the risk-of-bias assessment using the ROBINS-I tool (Sterne et al., 2016).

The overall judgment of the risk of bias was rated as serious in 40 (80%) studies and moderate in 10 (20%) studies (Fig. 2). The high proportion of studies judged as having a serious overall risk of bias was in the majority of cases due to the ‘Bias due to confounding’ domain being rated as having a serious risk.

### 3.3 Baseline characteristics

The 50 studies comprised 2328 patients. The weighted mean age was 42 years (pooled SD ± 14) and 47% (1097/2315) were women. Symptomatic presentations occurred in 95% (2159/2274) of patients, while 5% (115/2274) presented incidentally (Table 2). In 39 studies reporting the type of symptoms in symptomatic patients, most patients had motor symptoms (61% [816/1343]) and/or sensory symptoms (67% [903/1343]). Types of clinical presentations are provided in Supplemental Table 5. The location of SCM at the spinal cord was mostly cervical (41% [949/2328]) or thoracic (41% [961/2328]). SCM locations across the spinal cord are visualized in Supplemental Fig. 2. The location in the horizontal plane was superficial in 69% (255/368) and deep in 31% (113/368) of cases. The weighted mean diameter of SCM was 11 mm (pooled SD ± 6).

**Table 2:** Synthesized baseline characteristics of all patients and stratified by treatment strategy.

Variable	All patients (n = 2,328, 50 studies)	Surgical treatment (n = 1,802, 42 studies)	Conservative management (n = 374, 13 studies)	P-value
**Age (years)**	42 (±14)	42 (±14)	43 (±15)	0.82
**Sex**				
**Female**	1097 (47%)	850 (48%)	170 (45%)	0.47
**Male**	1218 (53%)	939 (52%)	204 (55%)	
**Mode of presentation**				
**Asymptomatic**	115 (5%)	76 (4%)	34 (9%)	**<0.001**
**Symptomatic**	2159 (95%)	1672 (96%)	340 (91%)	
**Motor**	816 (61%)	669 (68%)	81 (40%)	**<0.001**
**Sensory**	903 (67%)	673 (69%)	135 (66%)	0.45
**Pain**	438 (33%)	314 (32%)	80 (39%)	0.05
**Bladder or bowel**	292 (22%)	245 (25%)	27 (13%)	**<0.001**
**Gait disturbance**	55 (4%)	27 (3%)	18 (9%)	**<0.001**
**Location of SCM**				
**At spinal cord**				
**Cervical**	949 (41%)	736 (41%)	160 (43%)	0.49
**Cervicothoracic**	25 (1%)	19 (1%)	5 (1%)	0.59
**Thoracic**	961 (41%)	753 (42%)	116 (31%)	**<0.001**
**Thoracic or lumbar** **orthoracolumbar**	349 (15%)	269 (15%)	80 (21%)	**0.002**
**Lumbar or conus medullaris**	44 (2%)	25 (1%)	13 (3%)	**0.005**
**In horizontal plane**				
**Superficial**	255 (69%)	250 (70%)	5 (38%)	**0.027**
**Deep**	113 (31%)	105 (30%)	8 (62%)	
**Diameter of SCM (mm)**	11 (±6)	11 (±5)	10 (±7)	**0.003**

Data are weighted mean (±pooled SD) or number (%); p-values in bold were considered statistically significant (p < 0.05); study-level data and additional syntheses are shown in Supplemental Table 4, including the four studies (n = 152) that lacked baseline characteristics stratified by treatment strategy; numbers of patients and studies available for each variable are as follows: weighted mean age n = 2246 in 48 studies (pooled SD n = 1878 in 40 studies), sex n = 2315 in 49 studies, mode of presentation n = 2274 in 48 studies, type of symptoms among symptomatic patients n = 1343 in 39 studies (n = 982 for surgically treated patients and n = 205 for conservatively managed patients), location of SCM at spinal cord n = 2328 in 50 studies, location of SCM in horizontal plane n = 368 in 5 studies, weighted mean diameter of SCM n = 1286 in 25 studies (pooled SD n = 894 in 20 studies); the sum of the types of symptoms exceeds the number of symptomatic patients as multiple different symptoms could have occurred simultaneously; SCM, spinal cavernous malformation; SD, standard deviation.

Overall, 82% (1909/2328) of patients in 46 studies underwent surgical treatment, whereas 18% (419/2328) of patients across 17 studies underwent conservative management (13 studies reported on patients from either treatment group). Baseline characteristics stratified by treatment strategy are synthesized in Table 2, based on study-level data in Supplemental Table 4. Four studies did not provide stratified baseline characteristics (Badhiwala et al., 2014; Shan et al., 2002; Labauge et al., 2008; Früh et al., 2025). Demographics were comparable between surgically and conservatively treated patients (weighted mean age 42 years [pooled SD ± 14] versus 43 years [±15], p = 0.82, and proportion of women 48% [850/1789] versus 45% [170/374], p = 0.47).

Patients treated surgically were more often symptomatic (96% [1672/1748] versus 91% [340/374], p < 0.001), and more frequently had motor and bladder or bowel symptoms (68% [669/982] versus 40% [81/205], p < 0.001, and 25% [245/982] versus 13% [27/205], p < 0.001, respectively) than those managed conservatively. Additionally, surgically treated patients more frequently had their SCM located at the thoracic spinal cord (42% [753/1802] versus 31% [116/374], p < 0.001), whereas caudal locations were more common in conservatively managed patients (thoracic/lumbar or thoracolumbar 15% [269/1802] versus 21% [80/374], p = 0.002, and lumbar or conus medullaris 1% [25/1802] versus 3% [13/374], p = 0.005). Furthermore, the location of SCM in the horizontal plane was less often deep (30% [105/355] versus 62% [8/13], p = 0.027) and SCMs were larger (weighted mean SCM diameter 11 mm [pooled SD ± 5] versus 10 mm [±7], p = 0.003) in patients treated surgically versus conservatively.

### 3.4 Hemorrhage rates

The pooled annual rate of hemorrhage without or before surgery in which follow-up was censored after the first bleed could be calculated for 167 patients in 4 studies (649 person-years [mean 3.9 years] of follow-up, of whom 142 [85%] had initially presented with symptoms and 108 [65%] with a hemorrhage Supplemental Table 6). Thirty-one patients experienced a hemorrhage during follow-up, yielding a pooled annual rate of 4.8% (95% CI 3.2%-6.8%). The pooled annual incidence rate of hemorrhages during uncensored follow-up without or before surgery could be calculated for 226 patients in 4 studies (793 person-years [mean 3.5 years] of follow-up, 215 [95%] had initially presented with symptoms and 137 [61%] with a hemorrhage). Fifty hemorrhages occurred during follow-up, yielding a pooled annual rate of 6.3% (95% CI 4.7%-8.3%). Annual hemorrhage rates of the individual studies used in the latter calculation were higher if mean follow-up durations were shorter. Pooled annual hemorrhage rates calculated from birth, derived from 10 studies that assumed SCMs were congenital, are presented in Supplemental Table 6.

### 3.5 Outcomes of surgical treatment

Study-level data of outcomes in patients who underwent surgical treatment are provided in Supplemental Table 7. The weighted mean duration of symptoms was 21 months (pooled SD ± 36). Only one study reported the interval from presentation to surgery (median 32 days) (Kurokawa et al., 2023). Intraoperative neuromonitoring (IONM) was used in 98% (1358/1392) of patients. Complete resection was achieved in 94% (1641/1737), while the remainder underwent subtotal or partial resection, or biopsy. Postoperative complications occurred in 4% (47/1165, details in Supplemental Table 7). Postoperative (re)hemorrhage was experienced by 2% (9/590, all from a residual lesion).

**Fig. 3:**
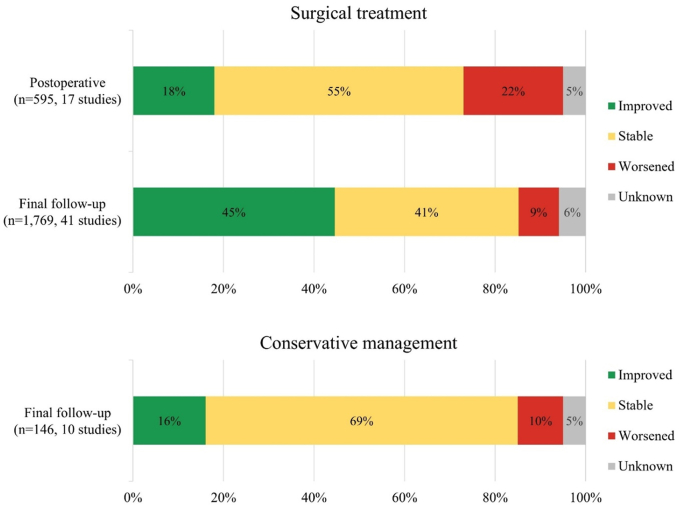
Distribution of the neurological outcome stratified by treatment strategy.

**Fig. 4:**
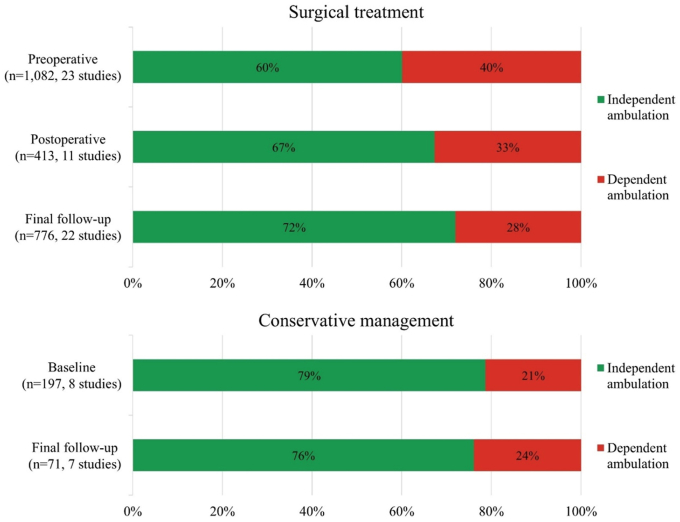
Proportions of patients who ambulated independently or not stratified by treatment strategy.

Compared to preoperatively, the postoperative neurological outcome had improved in 18% (109/595), was stable in 55% (325/595), had worsened in 22% (130/595), and was unknown in 5% (31/595) of patients (Fig. 3). The proportion of patients who ambulated independently postoperatively was 67% (278/413) versus 60% (650/1082) preoperatively (Fig. 4, based on study-level data in Supplemental Table 8 with synthesized distributions of each standardized outcome tool in Supplemental Fig. 3). The weighted mean postoperative follow-up was 43 months (pooled SD ± 38). At final follow-up, the neurological outcome had improved in 45% (788/1769), was stable in 41% (718/1769), had worsened in 9% (165/1769), and was unknown in 6% (98/1769) of patients, and 72% (559/776) ambulated independently.

### 3.6 Outcomes of conservative management

Study-level data of outcomes in patients who were managed conservatively are provided in Supplemental Table 9. The weighted mean follow-up was 47 months (pooled SD ± 34). Two studies reporting on surgically and conservatively treated patients did not provide outcomes for the conservative group (Shan et al., 2002; Nagoshi et al., 2019).

The final follow-up neurological outcome had improved in 16% (24/146), was stable in 69% (101/146), had worsened in 10% (14/146), and was unknown in 5% (7/146) of patients (Fig. 3). If the results of Ren et al. (2022) are included (who combined improved and stable outcomes), the neurological outcome had improved or was stable in 76% (208/272), had worsened in 21% (57/272), and was unknown in 3% (7/272) of patients. The proportion of patients who ambulated independently at final follow-up was 76% (54/71) versus 79% (155/197) at baseline (Fig. 4, based on study-level data in Supplemental Table 10 with synthesized distributions of each standardized outcome tool in Supplemental Fig. 3).

### 3.7 Comparative cohort analysis

Among the 13 studies reporting on both treatment groups (n = 517), the overall risk of bias was serious in 4 (31%) studies and moderate in 9 (69%) studies. Surgical treatment was performed in 63% (325/517) of patients, whereas 37% (192/517) were managed conservatively. In 9 studies that provided stratified characteristics, patients treated surgically were younger (weighted mean age 40 years [pooled SD ± 15] versus 48 years [±16], p < 0.001) and more often symptomatic (99% [215/218] versus 85% [125/147], p < 0.001) than those managed conservatively.

Postoperatively, the neurological outcome of surgically treated patients had improved in 28% (19/69), was stable in 42% (29/69), had worsened in 23% (16/69), and was unknown in 7% (5/69) of patients (Supplemental Table 11). The proportion of patients who ambulated independently was 74% (95/129) versus 58% (146/250) preoperatively (Supplemental Table 12). At final follow-up after surgical treatment, the neurological outcome had improved in 47% (112/239), was stable in 36% (85/239), had worsened in 12% (28/239), and was unknown in 6% (14/239) of patients, and 70% (174/250) ambulated independently. At final follow-up after conservative management, the neurological outcome had improved in 15% (21/140), was stable in 72% (101/140), had worsened in 8% (11/140), and was unknown in 5% (7/140) of patients, and 76% (54/71) ambulated independently versus 69% (49/71) at baseline.

## 4 Discussion

This systematic review of the literature on the treatment of patients with SCM shows that the majority of studies had a retrospective, single-center design and lacked a control arm, resulting in most reported cases being treated surgically, with outcomes often determined by the treating physicians. This suggests limited methodological quality of the included studies, as reflected by the serious overall risk of bias in the majority of studies. Additionally, the treatment groups may not have been directly comparable, as preoperatively, surgically treated patients less frequently had the ability to ambulate independently and were symptomatic more often than those managed conservatively at baseline. Conclusions regarding the optimal treatment should thus be drawn with caution, although surgery more frequently resulted in an improved neurological outcome at final follow-up.

Treatment decisions for patients with SCM are hampered by the rarity of the disorder and the resulting scarcity of studies. Previous systematic reviews aimed to settle the clinical equipoise by meta-analyzing surgical treatment versus conservative management and concluded that surgery was the preferred treatment (Badhiwala et al., 2014; Fotakopoulos et al., 2021; Rios-Zermeno et al., 2025). However, meta-analysis is not appropriate because the included studies were mostly single-center case series lacking a control group (Badhiwala et al., 2014), or were heterogeneous in terms of selection criteria, study outcomes, surgical techniques, and follow-up (Fotakopoulos et al., 2021; Rios-Zermeno et al., 2025), limiting the validity of pooled estimates. This is supported by the current review, as is demonstrated by the serious risk of bias in the majority of studies. Due to these limitations, we performed a descriptive analysis as well as a comparative cohort analysis, further demonstrating a difference in baseline disease severity between treatment groups that appears to persist even in direct comparative studies.

To distinguish treatment effects from the effects of patient selection, baseline characteristics stratified by treatment strategy are crucial. However, previous literature syntheses have not always provided this information (Asimakidou et al., 2022; Badhiwala et al., 2014), complicating the assessment of group comparability and evaluation of treatment effects. This issue is commonly observed in the literature, as four of our included studies, all involving patients treated either surgically or conservatively, also lacked stratified characteristics (Badhiwala et al., 2014; Shan et al., 2002; Labauge et al., 2008; Früh et al., 2025). In studies that did provide stratified characteristics, we found that demographics were comparable, but surgically treated patients were more frequently symptomatic, had larger SCMs, and had more often superficial SCM than those managed conservatively. These baseline differences further complicate comparisons of treatment outcomes and support our decision to refrain from performing meta-analysis. Furthermore, although complete resection was achieved in the majority of cases, selection bias may be present as particularly surgically treated SCMs are located superficial or dorsal, whereas deep or ventral lesions are managed conservatively more often and might have lower rates of complete resection (Akers et al., 2025). Surgical outcomes of deep SCMs remain unknown, even though their location is often a key reason for choosing conservative management to avoid (further) spinal cord damage.

Since surgically treated patients preoperatively less frequently ambulated independently and were more often symptomatic than conservatively managed patients at baseline, patients who presented with more severe disease may have been more likely to be treated surgically. This could introduce a bias against surgery, as these patients may have had a higher risk of poor outcomes regardless of treatment. However, the neurological outcome was measured as a change and was defined as improved, stable, or worsened. Therefore, patients with poorer outcomes at baseline may have had a higher chance to improve compared to conservatively managed patients who already had more favorable outcomes at baseline. Moreover, patients with severe disease who were treated surgically and experienced improvement might have also improved with conservative management, since the natural course of SCMs could result in improvement regardless of intervention, as has been observed in patients with CCM (Sandmann et al., 2025). Without any adjustment for confounding factors such as baseline disease severity, it is impossible to attribute improvements to the effect of treatment, as improvement might reflect natural recovery rather than true treatment effects.

We further found a discrepancy in the number of patients who were treated surgically or conservatively across the included studies and previously published systematic reviews (Asimakidou et al., 2022; Badhiwala et al., 2014), with the majority of cases being treated surgically. This has been suggested as being a reflection of general practice (Badhiwala et al., 2014; Fotakopoulos et al., 2021), but another plausible explanation is the selective publication of (favorable) outcomes after surgery. The majority of studies were retrospective and single-center, and lacked a control group of patients who were managed conservatively, which may indicate that the outcomes were reported by the physician who performed the treatment, while such favorable outcomes might have also occurred with conservative management. Meanwhile, data on postoperative complications and (re)bleeding were inconsistently reported. Studies often reported neurological outcomes and postoperative complications separately, while postoperative neurological worsening should be considered a complication. Since 22% of surgically treated patients had worsened directly postoperatively, the 4% complication rate may not be accurate. Furthermore, only two comparative studies specified whether outcomes were analyzed according to the treatment strategy assigned at baseline or by the treatment eventually performed (Ohnishi et al., 2020; Goyal et al., 2019). The remaining studies did not specify this and were presumably analyzed according to the ultimately applied strategy, despite influence of the chosen analytic approach on the interpretation of treatment effects, particularly if many conservatively managed patients later underwent surgery, as their outcomes may differ systematically.

The pooled annual risk of hemorrhage under observation was approximately 5%. We calculated pooled rates from presentation to capture the actual period during which patients are known to be at risk. These rates can be used to weigh against the up-front risks of surgical intervention and better inform treatment decisions than rates calculated from birth, a method used in ten studies and a previous meta-analysis (Badhiwala et al., 2014). Rates calculated from birth are often considered inaccurate and clinically irrelevant, as SCMs are neither diagnosed nor known to be at risk of bleeding from birth, thereby overestimating person-years as denominator and diluting the true rate. Nonetheless, hemorrhage rates from presentation should also be interpreted with caution, as they may reflect the rate of recurrent hemorrhage after an often-hemorrhagic presentation, although data on which hemorrhages occurred recurrently were lacking. Such rates should not be extrapolated to patients’ lifetimes, as risks may vary over time and tend to be higher during the initial years after a first hemorrhage, a pattern previously observed in patients with CCM (Sandmann et al., 2025). This is supported by the finding that annual hemorrhage incidence rates were lower in studies with longer mean follow-up periods. Furthermore, the presented hemorrhage rates refer to symptomatic events, whereas silent bleeds are increasingly detected with advancements in MRI, though the current data do not support recommendations for the management of such cases.

The clinical course of SCM may be more aggressive than that of CCMs. Our pooled per-patient annual hemorrhage rate from SCM of 4.8% was slightly higher than the 3.7% rate reported in a study of patients with conservatively managed CCMs (Sandmann et al., 2024). Additionally, the annual hemorrhage rate during overall follow-up was 6.3%, as compared to 2.3% in another study of patients with CCM (Flemming et al., 2012). Explanations may be the eloquence of the spinal cord and the limited space to compensate for hemorrhages (Maslehaty et al., 2011).

Strengths of this systematic review include the comprehensive search strategy, structured risk-of-bias assessment, and descriptive analysis of (the comparability of) surgical treatment versus conservative management. Several limitations should also be acknowledged. First, the included studies may not represent the complete population of patients with SCM, as most studies were single-center and conducted in tertiary care or neurosurgical referral centers, introducing selection bias. Second, all but two studies were retrospective, a design often associated with missing data, confounding factors, and recall and selection biases. Third, all studies were conducted in Western and East Asian countries, limiting generalizability to other regions. Fourth, the reporting of many variables was highly inconsistent across the included studies, particularly in the approach to calculating annual hemorrhage rates, and thus this rate should be interpreted with caution. Fifth, due to heterogeneity across studies, data were missing for several outcomes (e.g. the reasons for performing surgical intervention), which was addressed by consistently reporting the number of patients and studies available for each variable.

The current literature synthesis provides an overview of clinical outcomes following surgical treatment or conservative management of patients with SCM. We descriptively analyzed the available literature, acknowledging limitations and biases across the included studies, rather than attempting to meta-analyze the evidence, as it may result in misleading conclusions. Definitive conclusions of a benefit of surgical treatment versus conservative management are precluded due to the differences in disease severity between treatment groups, which complicate direct comparison, as well as low quality of studies and their high risk of bias. To better inform treatment decisions, future research should involve direct comparative studies, ideally in a randomized setting, using clinically relevant functional outcomes assessed in a blinded manner. Alternatively, long-term prospective, multicenter studies with propensity score matching of treatment groups may be conducted to increase comparability and reduce bias due to confounding. Establishing international registries for patients with SCM may be necessary to conduct such studies. Future research should also incorporate patient-reported outcomes and establish the natural history, providing additional data on the annual risk of hemorrhage from SCM.

## Author contributions

Abel Clemens Adriaan Sandmann: conceptualization, data curation, formal analysis, investigation, methodology, project administration, visualization, writing – original draft, and writing – review & editing. Marinus Abraham Kempeneers: conceptualization, data curation, investigation, methodology, validation, and writing – review & editing. K. Mariam Slot, René van den Berg, and William Peter Vandertop: writing – review & editing. Dagmar Verbaan and Jonathan M. Coutinho: conceptualization, methodology, resources, supervision, and writing – review & editing. All authors have approved the final article.

## Funding

This research did not receive any specific grant from funding agencies in the public, commercial, or not-for-profit sectors.

## Declaration of competing interest

The authors declare the following financial interests/personal relationships which may be considered as potential competing interests: Jonathan M. Coutinho reports a relationship with Bayer that includes: funding grants. Jonathan M. Coutinho reports a relationship with AstraZeneca that includes: funding grants. Jonathan M. Coutinho reports a relationship with TrianecT that includes: board membership and employment. René van den Berg reports a relationship with Johnson & Johnson that includes: consulting or advisory. If there are other authors, they declare that they have no known competing financial interests or personal relationships that could have appeared to influence the work reported in this paper.
