# Supplementary material for: Treatment of patients with spinal cavernous malformations: a systematic review
Source: Brain Spine. 2026 Mar 25;6:106021. doi: 10.1016/j.bas.2026.106021 (PMC13054064; doi:10.1016/j.bas.2026.106021)
Supplement: Multimedia component 1 [file mmc1.docx]

**Supplemental File**

Supplement to: *Treatment of patients with spinal cavernous malformations: a systematic review*

Supplemental Table 1. Systematic search strategy

| **Ovid MEDLINE(R) ALL 1946 to April 03, 2025** | | |
| --- | --- | --- |
| **#** | **Searches** | **Results** |
| 1 | exp Hemangioma, Cavernous/ and exp Spinal Cord/ | 139 |
| 2 | (SCCMs or SCVMs or ((Spinal or Intraspinal or vertebra* or medulla* or intramedulla* or backbone or spinalcord* or cervical* or dorsal* or sacrum or sacral or thoracic or lumbar or myelon or myelum or "medulla spinalis" or (lateral adj1 column) or (lateral adj1 cornu)) adj2 (cavernous or vascular or venous or "arterio venous") adj2 (malformation* or anomal* or angioma* or lesion* or Hemangioma*))).ti,ab,kf. | 1,122 |
| 3 | ((Spinal or Intraspinal or vertebra* or medulla* or intramedulla* or backbone or spinalcord* or cervical* or dorsal* or sacrum or sacral or thoracic or lumbar or myelon or myelum or "medulla spinalis" or (lateral adj1 column) or (lateral adj1 cornu)) adj2 cavernoma*).ti,ab,kf. | 145 |
| 4 | or/1-3 | 1,280 |
| 5 | exp conservative treatment/ | 5,793 |
| 6 | (untreat* or ((non or "not" or delay* or deferr* or postpone*) adj2 (operat* or therap* or treat* or surg* or intervent*))).ti,ab,kf. | 578,898 |
| 7 | ((conservativ* or nonsurg* or nonoperat* or nonintervent* or noninvasive or "non invasive" or "non surg*" or "non operat*" or "non intervent*") adj3 (therap* or treat* or manag* or observ* or approach* or care or surveill* or strateg*)).ti,ab,kf. | 171,407 |
| 8 | (Observation adj2 monitoring).ti,ab,kf. | 385 |
| 9 | (Watch* adj2 (wait* or observ* or mintor* or scan*)).ti,ab,kf. | 6,026 |
| 10 | ((Natural* or Unaltered or normal* or Untampered) adj2 (trajector* or development or course or progres*)).ti,ab,kf. | 54,353 |
| 11 | or/5-10 | 775,747 |
| 12 | exp General Surgery/ | 41,391 |
| 13 | (surger* or surgical* or operat* or intervention* or procedure* or resect* or excision or microcatheter* or emboli* or ((surgical or operati* or invasive or interventional or procedural or technical or practical) adj3 (treat* or therap* or procedure* or technique* or method* or manag* or process* or approach* or practice* or strateg* or modality or care or repair* or restorat* or correct* or expos* or servic* or specialt*))).ti,ab,kf. | 5,915,548 |
| 14 | ("dual lumen" adj2 balloon).ti,ab,kf. | 85 |
| 15 | (Laminectomy or Hemilaminectomy or Cordotomy or microsurg* or coagulat*).ti,ab,kf. | 175,456 |
| 16 | or/12-15 | 6,036,691 |
| 17 | 11 or 16 | 6,469,079 |
| 18 | 4 and 17 | 805 |
| 29 | case reports.pt. | 2,470,926 |
| 20 | 18 not 19 | 398 |
|  | | |
| **Ovid Embase Classic+Embase 1974 to 2025 April 03** | | |
| **#** | **Searches** | **Results** |
| 1 | exp cavernous hemangioma/ and exp spinal cord/ | 208 |
| 2 | (SCCMs or SCVMs or ((Spinal or Intraspinal or vertebra* or medulla* or intramedulla* or backbone or spinalcord* or cervical* or dorsal* or sacrum or sacral or thoracic or lumbar or myelon or myelum or "medulla spinalis" or (lateral adj1 column) or (lateral adj1 cornu)) adj2 (cavernous or vascular or venous or "arterio venous") adj2 (malformation* or anomal* or angioma* or lesion* or Hemangioma*))).ti,ab,kf. | 1,457 |
| 3 | ((Spinal or Intraspinal or vertebra* or medulla* or intramedulla* or backbone or spinalcord* or cervical* or dorsal* or sacrum or sacral or thoracic or lumbar or myelon or myelum or "medulla spinalis" or (lateral adj1 column) or (lateral adj1 cornu)) adj2 cavernoma*).ti,ab,kf. | 202 |
| 4 | or/1-3 | 1,708 |
| 5 | exp conservative treatment/ | 775,898 |
| 6 | (untreat* or ((non or "not" or delay* or deferr* or postpone*) adj2 (operat* or therap* or treat* or surg* or intervent*))).ti,ab,kf. | 856,402 |
| 7 | ((conservativ* or nonsurg* or nonoperat* or nonintervent* or noninvasive or "non invasive" or "non surg*" or "non operat*" or "non intervent*") adj3 (therap* or treat* or manag* or observ* or approach* or care or surveill* or strateg*)).ti,ab,kf. | 236,586 |
| 8 | (Observation adj2 monitoring).ti,ab,kf. | 571 |
| 9 | (Watch* adj2 (wait* or observ* or mintor* or scan*)).ti,ab,kf. | 9,708 |
| 10 | ((Natural* or Unaltered or normal* or Untampered) adj2 (trajector* or development or course or progres*)).ti,ab,kf. | 72,476 |
| 11 | or/5-10 | 1,792,243 |
| 12 | exp surgery/ | 6,440,184 |
| 13 | (surger* or surgical* or operat* or intervention* or procedure* or resect* or excision or microcatheter* or emboli* or ((surgical or operati* or invasive or interventional or procedural or technical or practical) adj3 (treat* or therap* or procedure* or technique* or method* or manag* or process* or approach* or practice* or strateg* or modality or care or repair* or restorat* or correct* or expos* or servic* or specialt*))).ti,ab,kf. | 7,735,562 |
| 14 | ("dual lumen" adj2 balloon).ti,ab,kf. | 108 |
| 15 | (Laminectomy or Hemilaminectomy or Cordotomy or microsurg* or coagulat*).ti,ab,kf. | 236,298 |
| 16 | or/12-15 | 10,753,122 |
| 17 | 11 or 16 | 11,694,364 |
| 18 | 4 and 17 | 1,344 |
| 29 | exp case report/ | 3,098,101 |
| 20 | 18 not 19 | 705 |
|  |  |  |
|  | | |
| **Web of Science Core Collection (WOS.SCI 1900-, WOS.AHCI 1975-, WOS.ESCI 2005-, WOS.SSCI 1956-2025)** | | |
| **#** | **Searches** | **Results** |
| 1 | TS=(SCCMs or SCVMs or ((Spinal or Intraspinal or vertebra* or medulla* or intramedulla* or backbone or spinalcord* or cervical* or dorsal* or sacrum or sacral or thoracic or lumbar or myelon or myelum or "medulla spinalis" or (lateral near/1 column) or (lateral near/1 cornu)) near/2 (cavernous or vascular or venous or "arterio venous") near/2 (malformation* or anomal* or angioma* or lesion* or Hemangioma*))) | 1,286 |
| 2 | TS=((Spinal or Intraspinal or vertebra* or medulla* or intramedulla* or backbone or spinalcord* or cervical* or dorsal* or sacrum or sacral or thoracic or lumbar or myelon or myelum or "medulla spinalis" or (lateral near/1 column) or (lateral near/1 cornu)) near/2 cavernoma*) | 168 |
| 3 | #1 OR #2 | 1,405 |
| 4 | TS=(untreat* or ((non or "not" or delay* or deferr* or postpone*) near/2 (operat* or therap* or treat* or surg* or intervent*))) | 880,550 |
| 5 | TS=((conservativ* or nonsurg* or nonoperat* or nonintervent* or noninvasive or "non invasive" or "non surg*" or "non operat*" or "non intervent*") near/3 (therap* or treat* or manag* or observ* or approach* or care or surveill* or strateg*)) | 176,164 |
| 6 | TS=(Observation near/2 monitoring) | 4,443 |
| 7 | TS=(Watch* near/2 (wait* or observ* or mintor* or scan*)) | 6,495 |
| 8 | TS=((Natural* or Unaltered or normal* or Untampered) near/2 (trajector* or development or course or progres*)) | 79,866 |
| 9 | #4 OR #5 OR #6 OR #7 OR #8 | 1,105,488 |
| 10 | TS=(surger* or surgical* or operat* or intervention* or procedure* or resect* or excision or microcatheter* or emboli* or ((surgical or operati* or invasive or interventional or procedural or technical or practical) near/3 (treat* or therap* or procedure* or technique* or method* or manag* or process* or approach* or practice* or strateg* or modality or care or repair* or restorat* or correct* or expos* or servic* or specialt*))) | 8,951,714 |
| 11 | TS=("dual lumen" near/2 balloon) | 92 |
| 12 | TS=(Laminectomy or Hemilaminectomy or Cordotomy or microsurg* or coagulat*) | 225,098 |
| 13 | #10 OR #11 OR #12 | 9,096,258 |
| 14 | #13 OR #9 | 9,734,913 |
| 15 | #14 AND #3 | 850 |
|  | | |
| **Central (Cochrane Library Wiley), date run 3-4-2025** | | |
| # | **Searches** | **Results** |
| 1 | MeSH descriptor: [Hemangioma, Cavernous] explode all trees | 28 |
| 2 | MeSH descriptor: [Spinal Cord] explode all trees | 1,172 |
| 3 | #1 AND #2 | 1 |
| 4 | (SCCMs or SCVMs or ((Spinal or Intraspinal or vertebra* or medulla* or intramedulla* or backbone or spinalcord* or cervical* or dorsal* or sacrum or sacral or thoracic or lumbar or myelon or myelum or "medulla spinalis" or (lateral near/1 column) or (lateral near/1 cornu)) near/2 (cavernous or vascular or venous or "arterio venous") near/2 (malformation* or anomal* or angioma* or lesion* or Hemangioma*))) | 3 |
| 5 | ((Spinal or Intraspinal or vertebra* or medulla* or intramedulla* or backbone or spinalcord* or cervical* or dorsal* or sacrum or sacral or thoracic or lumbar or myelon or myelum or "medulla spinalis" or (lateral near/1 column) or (lateral near/1 cornu)) near/2 cavernoma*) | 2 |
| 6 | #3 OR #4 OR #5 | 6 |

Supplemental Table 2. Descriptions of standardized scoring tools used in the literature

| **McCormick^1^ scale** | | | |
| --- | --- | --- | --- |
| **1** | Neurologically normal; mild focal deficit not significantly affecting function of involved limb; mild spasticity or reflex abnormality; normal gait | | |
| **2** | Presence of sensorimotor deficit affecting function of involved limb; mild to moderate gait difficulty; severe pain or dysesthesia impairing patient’s quality of life; still functions and ambulates independently | | |
| **3** | More severe neurological deficit; requires cane/brace for ambulation or significant bilateral upper extremity impairment; may or may not function independently | | |
| **4** | Severe deficit; requires wheelchair or cane/brace with bilateral upper extremity impairment; usually not independent | | |
|  | | | |
| **Modified McCormick^2^ scale** | | | |
| **1** | Neurologically intact, ambulates normally, may have minimal dysesthesia | | |
| **2** | Mild motor or sensory deficit, maintains functional independence | | |
| **3** | Moderate deficit, limitation of function, independent with external aid | | |
| **4** | Severe motor or sensory deficit, dependent with external aid | | |
| **5** | Paraplegia or quadriplegia | | |
|  | | | |
| **Frankel^3^ scale** | | | |
| **A** | Complete paralysis below the level of the lesion | | |
| **B** | Sensory function only below the level of the lesion | | |
| **C** | Non-functional motor function below the level of the lesion | | |
| **D** | Abnormal, functional motor function below the level of the lesion | | |
| **E** | Free of neurological symptoms; abnormal reflexes may be present | | |
|  | | | |
| **Aminoff-Logue Disability Scale^4^** | | | |
| **Gait** | | **Urination** | **Defecation** |
| 0: normal;  1: leg weakness and abnormal gait without activity restriction;  2: restricted activity without requiring support;  3: one stick required for walking;  4: two sticks or walker required for walking;  5: unable to stand, confined to wheelchair | | 0: normal;  1: continent with hesitancy, urgency, or altered sensation;  2: occasional urinary incontinence;  3: total incontinence | 0: normal;  1: moderate constipation;  2: severe constipation or occasional incontinence;  3: total incontinence |
| **I** | Normal with or without minor deficits and sphincter dysfunction (total score 0-2) | | |
| **II** | Moderate motor deficits or sphincter dysfunction with independent ambulation (total score 3-5) | | |
| **III** | Mildly severe neurological deficits and partial sphincter dysfunction with or without independent ambulation (total score 6-8) | | |
| **IV** | Severe neurological deficits and total sphincter dysfunction without independent ambulation (total score 9-11) | | |
|  | | | |
| **American Spinal Injury Association (ASIA) Impairment Scale^5^** | | | |
| **A** | No motor or sensory function is preserved in the sacral segments S4–S5 | | |
| **B** | Sensory but not motor function is preserved below the neurological level and includes the sacral segments S4–S5 | | |
| **C** | Motor function is preserved below the neurological level, and more than half of key muscles below the neurological level have a muscle grade less than 3 | | |
| **D** | Motor function is preserved below the neurological level, and at least half of key muscles below the neurological level have a muscle grade of 3 or more | | |
| **E** | Motor and sensory function is normal | | |

Supplemental Table 3. Articles excluded because their cohort overlapped with another included study

| **Authors and year** | **Journal** | **Country** | **Overlap with** |
| --- | --- | --- | --- |
| Vishteh *et al.* 1997 | Neurosurgery | USA | Srinivasan *et al.* 2023^6^ |
| Cristante *et al.* 1998 | Neurosurgery | Germany | Reitz *et al.* 2015^7^ |
| Huffmann *et al.* 1998 | Neurol Med Chir Suppl | Germany | Spetzger *et al.* 1995^8^ |
| Ghogawala *et al.* 1999 | Neurosurgery Cl N Am | USA | Ogilvy *et al.* 1992^9^ |
| Vishteh *et al.* 1999 | Neurosurgery | USA | Srinivasan *et al.* 2023^6^ |
| Zevgaridis *et al.* 1999 | Acta Neurochir | Germany | Steiger *et al.* 2010^10^ |
| Deutsch *et al.* 2000 | J Neurosurg | USA | Jallo *et al.* 2006^11^ |
| Kim *et al.* 2006 | Neurosurgery | USA | Srinivasan *et al.* 2023^6^ |
| Che *et al.* 2008 | Natl Med J China | China | Li *et al.* 2018^12^ |
| Matsumaya *et al.* 2009 | J Neurosurg Spine | Japan | Imagama *et al.* 2017^13^ |
| Lu *et al.* 2010 | Neurosurg Focus | USA | Srinivasan *et al.* 2023^6^ |
| Liang *et al.* 2011 | J Neurosurg Spine | China | Tian *et al.* 2023^14^ |
| Mitha *et al.* 2011 | J Neurosurg Spine | USA | Srinivasan *et al.* 2023^6^ |
| Tong *et al.* 2012 | J Neurosurg Spine | China | Zhang *et al.* 2016^15^ |
| Endo *et al.* 2013 | J Neurosurg Spine | Japan | Kurokawa *et al.* 2023^16^ |
| Qing *et al.* 2014 | Neurology India | China | Liao *et al.* 2022^17^ |
| Ardeshiri *et al.* 2016 | Neurosurg Rev | Germany | Santos *et al.* 2022^18^ |
| Ren *et al.* 2018 | Spine | China | Tian *et al.* 2023^14^ |
| Li *et al.* 2019 | World Neurosurgery | China | Tian *et al.* 2023^14^ |
| Ren *et al.* 2019 | J Neurosurg Spine | China | Tian *et al.* 2023^14^ |
| Ren *et al.* 2019 | Frontiers in Neurology | China | Tian *et al.* 2023^14^ |
| Li *et al.* 2020 | Chin J Contemp Neurol Nch | China | Tian *et al.* 2023^14^ |
| Ren *et al.* 2020 | Neurosurgery | China | Tian *et al.* 2023^14^ |
| Liu *et al.* 2021 | J Neurosurg Spine | China | Liu *et al.* 2023^19^ |
| Duan *et al.* 2022 | Frontiers in Medicine | China | Li *et al.* 2018^12^ |
| Pantel *et al.* 2022 | European Spine Journal | Germany | Reitz *et al.* 2015^7^ |
| Rauschenbach *et al.* 2023 | J Neurosurg Spine | Germany | Santos *et al.* 2022^18^ |
| Rauschenbach *et al.* 2023 | European Spine Journal | Germany | Santos *et al.* 2022^18^ |
| Rauscher *et al.* 2023 | Eur J Neurol | Germany | Santos *et al.* 2022^18^ |
| Santos *et al.* 2023 | Nature Scientific Reports | Germany | Santos *et al.* 2022^18^ |

Supplemental Table 4. Detailed characteristics of the fifty included studies stratified by treatment strategy

| **Studies that included patients who underwent surgical treatment** | | | | | | | | | | | | | | | | | | | | |
| --- | --- | --- | --- | --- | --- | --- | --- | --- | --- | --- | --- | --- | --- | --- | --- | --- | --- | --- | --- | --- |
| **Authors and year** | **N** | **Age (±SD)** | **F (%)** | **Ce** | **CT** | **Th** | **T/L** | **TL** | **Lu** | **CM** | **Su** | **De** | **D (±SD)** | **As** | **Sym** | **Mo** | **Se** | **Pn** | **BB** | **G** |
| Cosgrove *et al.* 1988^20^ | 5 | 41 (±8) | 3 (60) | 1 |  | 4 |  |  |  |  |  |  | 25 (±6) | 0 | 5 | 4 | 5 | 2 | 1 |  |
| McCormick *et al.* 1988^21^ | 6 | 33 (±16) | 2 (33) | 1 |  | 5 |  |  |  |  |  |  |  | 0 | 6 | 6 | 6 | 4 | 3 | 2 |
| Ogilvy *et al.* 1992^9^ | 6 | 42 (±8) | 3 (50) | 4 |  | 2 |  |  |  |  |  |  |  | 0 | 6 | 5 | 6 | 3 | 1 |  |
| Cantore *et al.* 1995^22^ | 6 | 54 (±7) | 1 (17) | 2 |  | 4 |  |  |  |  |  |  | 17 (±7) | 0 | 6 | 5 | 5 |  | 2 |  |
| Spetzger *et al.* 1995^8^ | 9 | 43 (±14) | 3 (33) | 4 |  | 4 |  | 1 |  |  |  |  |  | 0 | 9 | 9 | 5 |  | 4 | 6 |
| Padovani *et al.* 1997^23^ | 6 | 45 (±10) | 2 (33) | 2 |  | 3 |  |  | 1 |  |  |  |  | 0 | 6 | 4 | 5 | 2 | 1 |  |
| Tu *et al.* 1999^24^ | 7 | 30 (±19) | 2 (29) | 4 | 1 | 2 |  |  |  |  |  |  |  | 0 | 7 | 6 | 2 | 2 | 2 |  |
| Sandalcioglu *et al.* 2003^25^ | 10 | 38 (±16) | 7 (70) | 5 |  | 5 |  |  |  |  |  |  |  | 0 | 10 |  |  |  |  |  |
| Santoro *et al.* 2004^26^ | 10 | 41 (±19) | 5 (50) | 5 |  | 5 |  |  |  |  |  |  | 10 (±3) | 0 | 10 | 8 | 8 | 1 | 4 |  |
| Jallo *et al.* 2006^11^ | 26 | 38 (±16) | 9 (35) | 8 | 2 | 16 |  |  |  |  |  |  | 16 (8-45)^1^ | 0 | 26 | 15 | 7 | 4 | 2 |  |
| Kharkar *et al.* 2007^27^ | 4 | 45 (±8) | 2 (50) | 1 |  | 2 |  |  | 1 |  |  |  |  | 0 | 4 | 4 | 1 | 4 |  |  |
| Gu *et al.* 2008^28^ | 28 | 36 (12-52)^1^ | 16 (57) | 8 | 12 | 4 |  | 2 | 2 |  |  |  |  | 0 | 28 |  |  |  |  |  |
| Bian *et al.* 2009^29^ | 16 | 38 (±15) | 9 (56) | 8 | 1 | 7 |  |  |  |  |  |  | 5 (±3) | 0 | 16 | 8 | 14 | 5 | 2 |  |
| Park *et al.* 2009^30^ | 14 | 34 (±10) | 5 (36) | 4 |  | 7 |  |  |  | 3 |  |  |  | 0 | 14 | 9 | 14 |  |  |  |
| Deutsch 2010^31^ | 5 | 56 (±15) | 2 (40) | 1 |  | 4 |  |  |  |  |  |  |  | 0 | 5 |  |  | 5 |  |  |
| Steiger *et al.* 2010^10^ | 17 | 40 (±12) | 9 (53) | 6 |  | 11 |  |  |  |  |  |  | 16 (±11) | 0 | 17 | 9 | 10 |  | 2 | 2 |
| Aoyama *et al.* 2011^32^ | 12 | 33 (±16) | 5 (42) | 5 |  | 6 |  |  | 1 |  |  |  |  | 0 | 12 |  |  |  |  |  |
| Choi *et al.* 2011^2^ | 21 | 39 (±16) | 13 (62) | 10 |  | 9 |  | 1 | 1 |  |  |  | 12 (±5) | 0 | 21 | 13 | 13 | 10 | 4 | 2 |
| Maslehaty *et al.* 2011^33^ | 11 | 42 (±17) | 9 (82) | 5 |  | 4 |  |  |  | 2 |  |  |  | 0 | 11 | 3 | 9 | 6 | 1 | 3 |
| Wachter *et al.* 2012^34^ | 30 | 42^2^ (18-71)^1^ | 17 (57) | 10 |  | 19 |  | 1 |  |  |  |  |  | 3 | 27 | 10 | 25 |  | 8 |  |
| Li *et al.* 2014^35^ | 21 | 39 (12-64)^1^ | 8 (38) | 6 |  | 12 |  |  | 3 |  |  |  | 15 (5-62)^1^ | 0 | 21 | 10 | 16 | 3 | 4 |  |
| Reitz *et al.* 2015^7^ | 48 | 41 (±16) | 23 (48) | 19 |  | 27 |  | 2 |  |  |  |  |  | 3 | 45 |  |  |  |  |  |
| Zhang *et al.* 2016^15^ | 58 | 39 (±15) | 23 (40) | 27 |  | 29 |  |  | 2 |  | 35 | 23 | 13 (±5) | 0 | 58 | 46 | 43 | 23 | 19 |  |
| Imagama *et al.* 2017^13^ | 41 | 39 (20-72)^1^ | 23 (56) | 17 |  | 24 |  |  |  |  |  |  |  | ? | ? |  |  |  |  |  |
| Sun *et al.* 2017^36^ | 10 | 41 (±11) | 5 (50) | 6 |  | 4 |  |  |  |  |  |  |  | 0 | 10 |  |  |  |  |  |
| Azad *et al.* 2018^37^ | 32 | 44 (±17) | 19 (59) | 16 |  | 16 |  |  |  |  |  |  | 7 (±4) | 0 | 32 | 16 | 26 | 16 | 6 |  |
| Ghobrial *et al.* 2018^38^ | 13 | 51 (±14) | ? | 9 |  | 4 |  |  |  |  |  |  |  | ? | ? |  |  |  |  |  |
| Li *et al.* 2018^12^ | 83 | 39 (±15) | 43 (52) | 34 |  | 47 |  |  | 2 |  | 66 | 17 |  | 0 | 83 | 47 | 9 | 25 | 13 |  |
| Velz *et al.* 2018^39^ | 21 | 42 (±20) | 12 (57) | 8 | 1 | 12 |  |  |  |  |  |  |  | 1 | 20 | 13 | 15 |  | 8 |  |
| Goyal *et al.* 2019^40^ | 21 | 41 (±17) | 6 (29) | 6 |  | 15 |  |  |  |  |  |  | 11 (±6) | 1 | 20 | 6 | 4 | 3 | 6 |  |
| Nagoshi *et al.* 2019^41^ | 57 | 45 (±16) | 27 (47) | 23 |  | 34 |  |  |  |  |  |  | 6 (±4) | 0 | 57 | 37 | 51 | 16 | 15 |  |
| Ohnishi *et al.* 2020^42^ | 5 | 26 (9-49)^1^ | 2 (40) | 2 |  | 3 |  |  |  |  | 1 | 4 | 20 (±6) | 0 | 5 | 5 | 5 | 0 | 4 |  |
| Zhang *et al.* 2021^43^ | 111 | 41 (±13) | 51 (46) | 42 |  |  | 69 |  |  |  | 82 | 29 | 11 (±6) | 0 | 111 | 89 | 92 | 51 | 42 |  |
| Zhang *et al.* 2021^44^ | 16 | 13 (±5) | 6 (38) | 8 |  | 8 |  |  |  |  |  |  | 15 (±8) | 0 | 16 | 13 | 6 | 8 | 5 |  |
| Liao *et al.* 2022^17^ | 98 | 42 (±17) | 35 (36) | 50 |  | 45 |  |  | 3 |  | 66 | 32 | 12 (±5) | 17 | 81 | 35 | 53 | 46 | 16 |  |
| Niedermeyer *et al.* 2022^45^ | 17 | 41 (±14) | 9 (53) | 8 |  | 9 |  |  |  |  |  |  | 11 (±6) | 0 | 17 | 6 | 16 | 6 | 4 | 7 |
| Cai *et al.* 2023^46^ | 29 | 45 (±14) | 12 (41) | 14 |  | 15 |  |  |  |  |  |  | 10 (±4) | 0 | 29 | 4 | 5 | 4 | 11 | 5 |
| Chen *et al.* 2023^47^ | 19 | 49 (±17) | 9 (47) | 4 | 2 | 12 |  |  | 1 |  |  |  | 11 (±5) | 1 | 18 | 12 | 17 | 13 | 7 |  |
| Kurokawa *et al.* 2023^16^ | 160 | 52 (7-85)^1^ | 70 (44) | 73 |  | 87 |  |  |  |  |  |  |  | 0 | 160 |  |  |  |  |  |
| Liu *et al.* 2023^19^ | 268 | 48 (±10) | 141 (53) | 114 |  | 151 |  |  | 3 |  |  |  | 11 (3-26)^1^ | 50 | 218 | 212 | 180 | 52 | 48 |  |
| Srinivasan *et al.* 2023^6^ | 146 | 45 (±15) | 77 (53) | 70 |  | 76 |  |  |  |  |  |  | 11 (±5) | 0 | 146 |  |  |  |  |  |
| Tian *et al.* 2023^14^ | 279 | 37 (±15) | 125 (45) | 86 |  |  | 193 |  |  |  |  |  |  | 0 | 279 |  |  |  |  |  |
| **Synthesis** | **1,802** | **42 (±14)** | **48%** | **736** | **19** | **753** | **262** | **7** | **20** | **5** | **250** | **105** | **11 (±5)** | **76** | **1,672** | **669** | **673** | **314** | **245** | **27** |
|  | | | | | | | | | | | | | | | | | | | | |
| **Studies that included patients who underwent conservative management** | | | | | | | | | | | | | | | | | | | | |
| **Authors and year** | **N** | **Age (±SD)** | **F (%)** | **Ce** | **CT** | **Th** | **T/L** | **TL** | **Lu** | **CM** | **Su** | **De** | **D (±SD)** | **As** | **Sym** | **Mo** | **Se** | **Pn** | **BB** | **G** |
| Kharkar *et al.* 2007^27^ | 10 | 41 (±18) | 4 (40) | 3 |  | 6 |  |  | 1 |  |  |  |  | 0 | 10 | 5 | 7 | 4 | 1 |  |
| Steiger *et al.* 2010^10^ | 3 | 34 (±8) | 2 (67) | 1 | 1 |  |  |  | 1 |  |  |  | 15 | 0 | 3 |  | 3 |  |  | 1 |
| Hegde *et al.* 2012^48^ | 6 | 39 (±23) | 3 (50) | 4 | 2 | 0 |  |  |  |  |  |  | 9 (±8) | 1 | 5 | 4 | 3 | 1 |  |  |
| Kim *et al.* 2013^49^ | 24 | 52 (±4) | 11 (46) | 12 |  | 10 |  |  | 2 |  |  |  |  | 5 | 19 | 7 | 14 | 5 |  |  |
| Zhang *et al.* 2016^15^ | 27 | 44 (±12) | 11 (41) | 13 |  | 11 |  |  | 3 |  |  |  | 9 (±5) | 0 | 27 | 16 | 24 | 10 | 1 |  |
| Velz *et al.* 2018^39^ | 8 | 53 (±18) | 4 (50) | 2 | 2 | 4 |  |  |  |  |  |  |  | 2 | 6 | 4 | 5 |  | 3 |  |
| Goyal *et al.* 2019^40^ | 64 | 53 (±17) | 34 (53) | 31 |  | 33 |  |  |  |  |  |  | 10 (±8) | 19 | 45 | 8 | 10 | 12 | 9 |  |
| Nagoshi *et al.* 2019^41^ | 9 | 48 (±16) | 3 (33) | 6 |  | 3 |  |  |  |  |  |  | 3 (±2) | 0 | 9 |  |  |  |  |  |
| Ohnishi *et al.* 2020^42^ | 13 | 41 (15-68)^1^ | 5 (38) | 4 |  | 9 |  |  |  |  | 5 | 8 | 13 (±5) | 0 | 13 | 7 | 10 | 5 |  |  |
| Zhang *et al.* 2021^44^ | 2 | 16 (±1) | 0 (0) | 1 |  | 1 |  |  |  |  |  |  | 5 (±1) | 0 | 2 | 2 | 2 | 2 | 1 |  |
| Ren *et al.* 2022^50^ | 126 | 35 (±15) | 55 (44) | 46 |  |  | 80 |  |  |  |  |  | 10 (±7) | 0 | 126 |  |  |  |  |  |
| Santos *et al.* 2022^18^ | 71 | 44 (±14) | 31 (44) | 30 |  | 36 |  |  | 5 |  |  |  |  | 6 | 65 | 21 | 48 | 37 | 11 | 17 |
| Chen *et al.* 2023^47^ | 11 | 46 (±10) | 7 (64) | 7 |  | 3 |  |  | 1 |  |  |  | 14 (±7) | 1 | 10 | 7 | 9 | 4 | 1 |  |
| **Synthesis** | **374** | **43 (±15)** | **45%** | **160** | **5** | **116** | **80** |  | **13** |  | **5** | **8** | **10 (±7)** | **34** | **340** | **81** | **135** | **80** | **27** | **18** |
|  | | | | | | | | | | | | | | | | | | | | |
| **Studies that included patients who underwent surgical treatment or conservative management but lacked stratified baseline characteristics** | | | | | | | | | | | | | | | | | | | | |
| **Authors and year** | **N** | **Age (±SD)** | **F (%)** | **Ce** | **CT** | **Th** | **T/L** | **TL** | **Lu** | **CM** | **Su** | **De** | **D (±SD)** | **As** | **Sym** | **Mo** | **Se** | **Pn** | **BB** | **G** |
| Shan *et al.* 2002^51^ | 23 | 32 (15-62)^1^ | 10 (43) | 6 |  | 15 |  |  | 2 |  |  |  |  | 0 | 23 | 17 | 19 | 6 | 10 |  |
| Labauge *et al.* 2008^52^ | 53 | 40 (11-80)^1^ | 27 (51) | 12 |  | 41 |  |  |  |  |  |  | 16 (3-54)^1^ | 1 | 52 | 28 | 39 |  |  |  |
| Badhiwala *et al.* 2014^53^ | 24 | 40 (2-80)^1^ | 10 (42) | 10 | 1 | 13 |  |  |  |  |  |  | 11 | 0 | 24 | 8 | 14 | 12 | 4 |  |
| Früh *et al.* 2025^54^ | 17 | 54^2^ (43-60)^3^ | 30 (58) | 25 |  | 23 |  |  | 4 |  |  |  | 7^2^ (4-10)^3^ | 4 | 48 | 13 | 23 | 26 | 6 | 10 |
| **Synthesis** | **152** | **38 (2-80)^1^** | **51%** | **53** | **1** | **92** |  |  | **6** |  |  |  | **15 (3-54)^1^** | **5** | **147** | **66** | **95** | **44** | **20** | **10** |
|  | | | | | | | | | | | | | | | | | | | | |
| **Overall synthesis** | **2,328** | **42 (±14)** | **47%** | **949** | **25** | **961** | **342** | **7** | **39** | **5** | **255** | **113** | **11 (±6)** | **115** | **2,159** | **816** | **903** | **438** | **292** | **55** |

Data are number, mean (±SD), number (%), or otherwise specified; the weighted mean age was 42 years (n=2,246, 48 studies) with a pooled SD of ±14 years (n=1,878, 40 studies); 47% patients were women (1,097 out of 2,315, 49 studies); the weighted mean SCM diameter was 11 mm (n=1,286, 25 studies) with a pooled SD of ±6 mm (n=894, 20 studies); As, asymptomatic; BB, bladder or bowel symptoms; Ce, cervical; CM, conus medullaris; CT, cervicothoracic; D, diameter (mm); De, deep; F, female; Lu, lumbar; Mo, motor symptoms; N, number; Pn, pain; SCM, spinal cavernous malformation; SD, standard deviation; Se, sensory symptoms; Su, superficial; Sym, symptomatic; Th, thoracic; T/L, thoracic or lumbar; TL, thoracolumbar.

^1^Range

^2^Median

^3^Interquartile range (IQR)


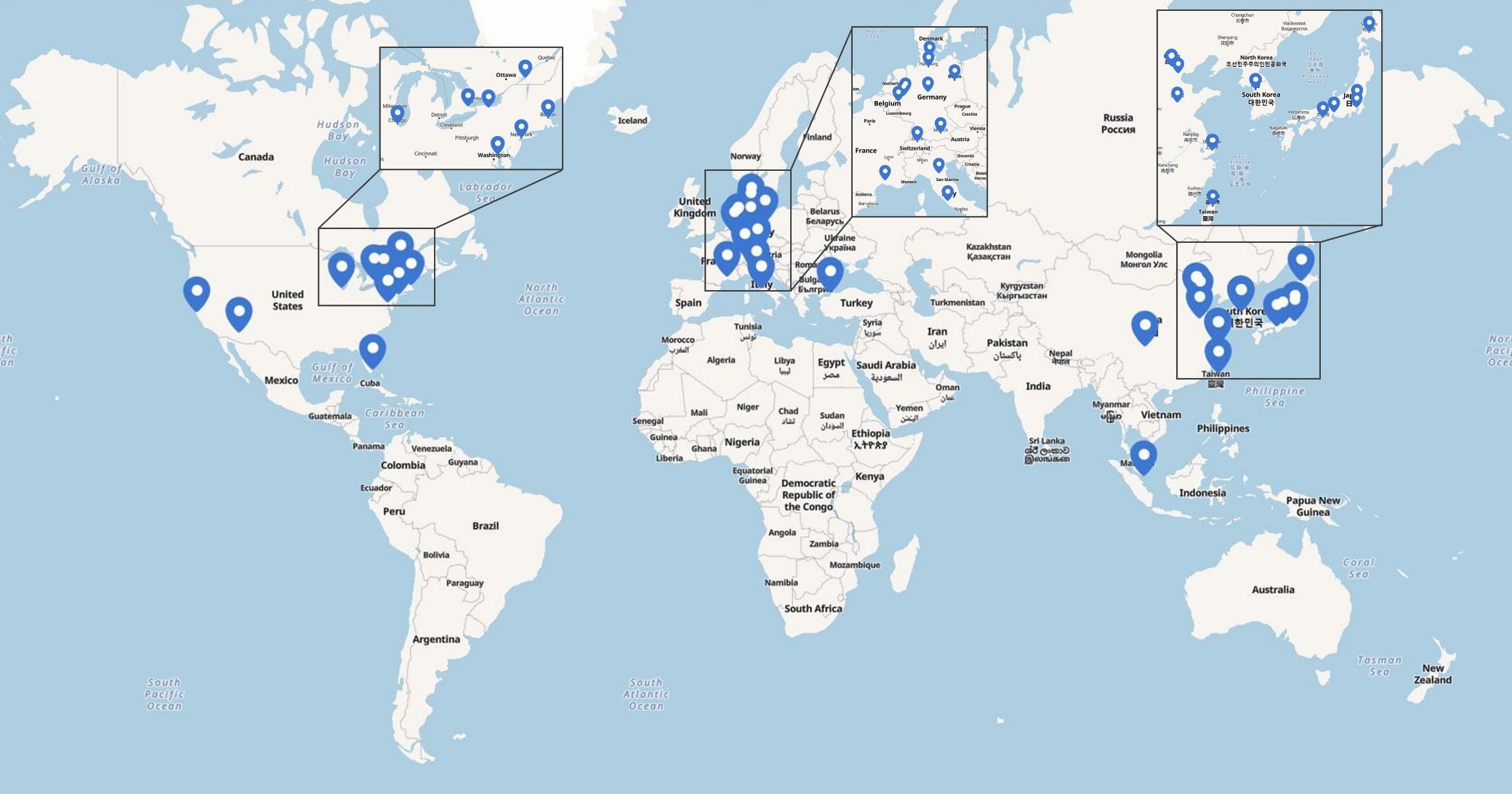


Supplemental Figure 1. World map depicting the locations where the fifty included studies were conducted

Supplemental Table 5. Mode of presentation and clinical course of symptomatic patients

| **Authors and year** | **N** | **Acute** | | | | | | **Progressive** | | | | |
| --- | --- | --- | --- | --- | --- | --- | --- | --- | --- | --- | --- | --- |
| Cosgrove *et al.* 1988^20^ | 5 | 2 | | | | | | 3 | | | | |
| McCormick *et al.* 1988^21^ | 6 | 1 | | | | | | 5 | | | | |
| Spetzger *et al.* 1995^8^ | 9 | 3 | | | | | | 6 | | | | |
| Padovani *et al.* 1997^23^ | 6 | 4 | | | | | | 2 | | | | |
| Tu *et al.* 1999^24^ | 7 | 5 | | | | | | 2 | | | | |
| Shan *et al.* 2002^51^ | 22 | 8 | | | | | | 14 | | | | |
| Sandalcioglu *et al.* 2003^25^ | 10 | 0 | | | | | | 10 | | | | |
| Jallo *et al.* 2006^11^ | 26 | 14 | | | | | | 12 | | | | |
| Gu *et al.* 2008^28^ | 28 | 6 | | | | | | 22 | | | | |
| Labauge *et al.* 2008^52^ | 52 | 32 | | | | | | 20 | | | | |
| Bian *et al.* 2009^29^ | 16 | 13 | | | | | | 3 | | | | |
| Park *et al.* 2009^30^ | 14 | 7 | | | | | | 7 | | | | |
| Steiger *et al.* 2010^10^ | 20 | 13 | | | | | | 7 | | | | |
| Maslehaty *et al.* 2011^33^ | 11 | 9 | | | | | | 2 | | | | |
| Hegde *et al.* 2012^48^ | 5 | 3 | | | | | | 2 | | | | |
| Badhiwala *et al.* 2014^53^ | 24 | 11 | | | | | | 13 | | | | |
| Li *et al.* 2014^35^ | 21 | 7 | | | | | | 14 | | | | |
| Velz *et al.* 2018^39^ | 26 | 16 | | | | | | 10 | | | | |
| Zhang *et al.* 2021^44^ | 18 | 7 | | | | | | 11 | | | | |
| Niedermeyer *et al.* 2022^45^ | 17 | 8 | | | | | | 9 | | | | |
| Srinivasan *et al.* 2023^6^ | 44 | 41 | | | | | | 3 | | | | |
| **Synthesis** | **387** | **177 (46%)** | | | | | | **210 (54%)** | | | | |
|  | | | | | | | | | | | | |
| **Authors and year** | **N** | **Ogilvy^1^** | | | | | | | | | | |
|  |  | **1** | | **2** | | | **3** | | | | **4** | |
| Ogilvy *et al.* 1992^9^ | 6 | 4 | | 2 | | | 0 | | | | 0 | |
| Cantore *et al.* 1995^22^ | 6 | 2 | | 4 | | | 0 | | | | 0 | |
| Santoro *et al.* 2004^26^ | 10 | 3 | | 5 | | | 2 | | | | 0 | |
| Kharkar *et al.* 2007^27^ | 6 | 3 | | 2 | | | 1 | | | | 0 | |
| Aoyama *et al.* 2011^32^ | 12 | 5 | | 2 | | | 2 | | | | 3 | |
| Reitz *et al.* 2015^7^ | 45 | 16 | | 9 | | | 8 | | | | 12 | |
| Sun *et al.* 2017^36^ | 10 | 1 | | 6 | | | 2 | | | | 1 | |
| Azad *et al.* 2018^37^ | 32 | 9 | | 13 | | | 6 | | | | 4 | |
| Goyal *et al.* 2019^40^ | 65 | 21 | | 12 | | | 11 | | | | 21 | |
| Nagoshi *et al.* 2019^41^ | 57 | 18 | | 25 | | | 3 | | | | 11 | |
| Zhang *et al.* 2021^43^ | 111 | 16 | | 62 | | | 29 | | | | 4 | |
| Liu *et al.* 2023^19^ | 218 | 31 | | 35 | | | 95 | | | | 57 | |
| **Synthesis** | **578** | **129 (22%)** | | **177 (31%)** | | | **159 (28%)** | | | | **113 (20%)** | |
|  | | | | | | | | | | | | |
| **Authors and year** | **N** | **Modified Ogilvy^2^** | | | | | | | | | | |
|  |  | **1** | **2** | | | **3** | | | **4** | | | **5** |
| Ren *et al.* 2022^50^ | 126 | 48 | 16 | | | 7 | | | 38 | | | 17 |
| Tian *et al.* 2023^14^ | 279 | 71 | 65 | | | 32 | | | 90 | | | 21 |
| **Synthesis** | **405** | **119 (29%)** | **81 (20%)** | | | **39 (10%)** | | | **128 (32%)** | | | **38 (9%)** |
|  | | | | | | | | | | | | |
| **Authors and year** | **N** | **Choi^3^** | | | | | | | | | | |
|  |  | **Type A** | | | **Type C** | | | | | **Type M** | | |
| Choi *et al.* 2011^2^ | 21 | 8 | | | 8 | | | | | 5 | | |
| Zhang *et al.* 2016^15^ | 85 | 17 | | | 44 | | | | | 24 | | |
| Cai *et al.* 2023^46^ | 29 | 12 | | | 15 | | | | | 2 | | |
| **Synthesis** | **135** | **37 (27%)** | | | **67 (50%)** | | | | | **31 (23%)** | | |

Data are number or number (%).

^1^Ogilvy^9^: (1) episodes of neurological deterioration with in-between recovery; (2) slow progression of neurological decline; (3) acute onset of symptoms with rapid decline; (4) acute onset of mild symptoms with subsequent gradual decline

^2^Modified Ogilvy^55^: (1) episodes of neurological deterioration with in-between recovery; (2) acute onset of symptoms with rapid decline; (3) acute onset of mild symptoms with progressive worsening; (4) acute onset of mild symptoms with stable asymptomatic, transient, or minimally symptomatic status; (5) slow progression of neurological decline

^3^Choi^2^: (type A) acute onset of symptoms with rapid decline; (type C) slow progression of neurological degeneration; (type M) acute neurological decline from various sequelae due to previous hemorrhages


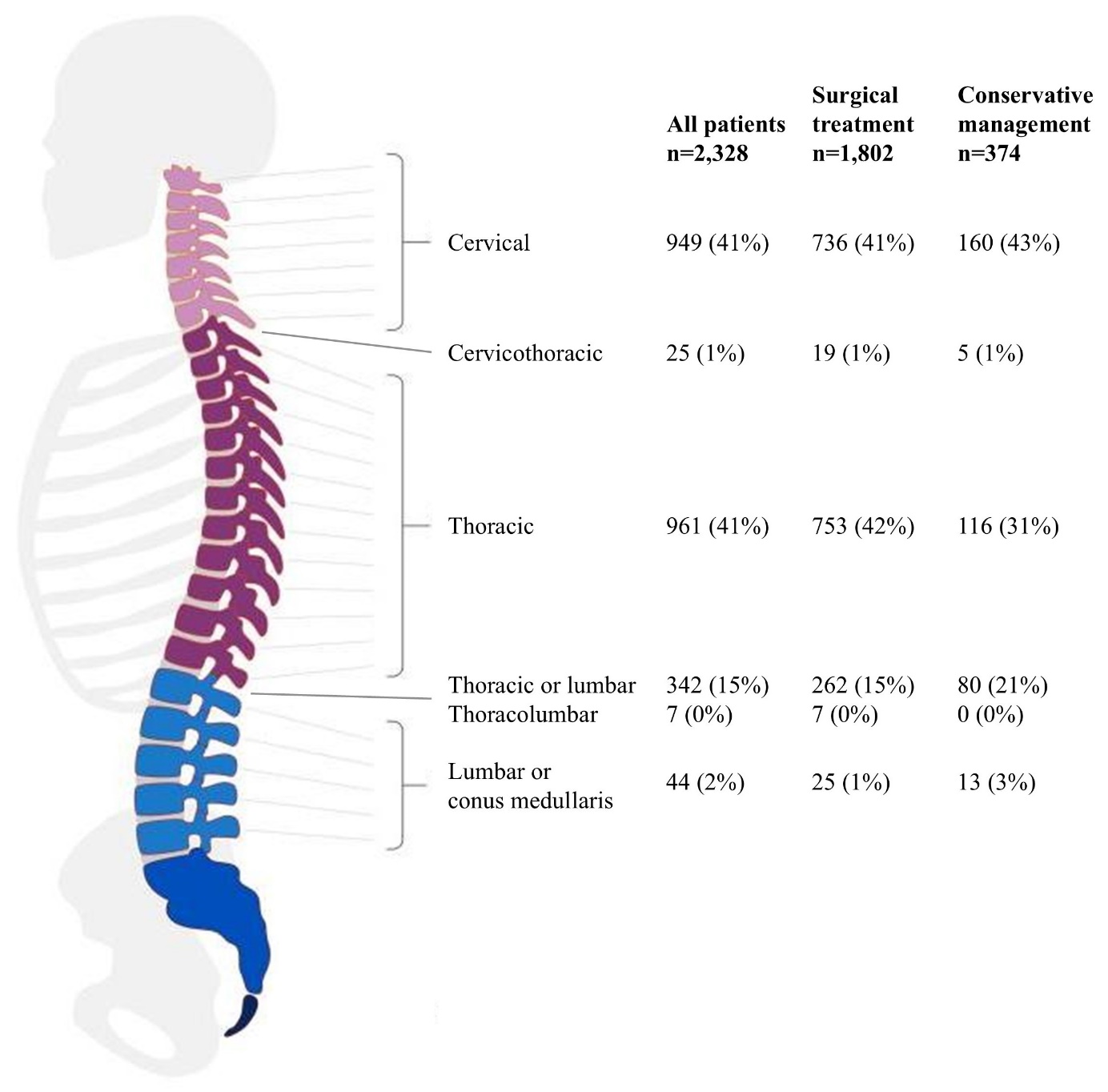


Supplemental Figure 2. SCM locations at the spinal cord among all patients and stratified by treatment strategy; in the four studies that lacked baseline characteristics stratified by treatment strategy (n=152), locations of SCM were cervical (n=53), cervicothoracic (n=1), thoracic (n=92), and lumbar (n=6)

Supplemental Table 6. Pooled annual hemorrhage rates after presentation or from birth

| **Pooled annual rate of hemorrhage after presentation during follow-up (with censoring)** | | | | | | | | | |
| --- | --- | --- | --- | --- | --- | --- | --- | --- | --- |
| **Authors and year** | **N** | **As** | | **Hx** | **FU** | **N hem-orrhage** | | **Person-years** | **Annual hemorrhage rate (95% CI)** |
| Deutsch 2010^31^ | 5 | 0 | | 0 | 5.2 | 0 | | 26 | 0.0% (0.0%-1.4%) |
| Zhang *et al.* 2016^15^ | 27 | 0 | | 27 | 2.8 | 3 | | 76 | 3.9% (0.8%-11.5%) |
| Goyal *et al.* 2019^40^ | 64 | 19 | | 37 | 4.5 | 16 | | 290 | 5.5% (3.2%-9.0%) |
| Santos *et al.* 2022^18^ | 71 | 6 | | 44 | 3.6 | 12 | | 257 | 4.7% (2.4%-8.2%) |
| **Synthesis** | **167** | **25** | | **108** | **3.9** | **31** | | **649** | **4.8% (3.2%-6.8%)** |
|  | | | | | | | | | |
| **Pooled annual rate of all hemorrhages after presentation during follow-up** | | | | | | | | | |
| **Authors and year** | **N** | **As** | | **Hx** | **FU** | **Hemo-rrhages** | | **Person-years** | **Annual hemorrhage rate (95% CI)** |
| Deutsch 2010^31^ | 5 | 0 | | 0 | 5.2 | 0 | | 26 | 0.0% (0.0%-1.4%) |
| Kim *et al.* 2013^49^ | 24 | 5 | | 0 | 5.0 | 2 | | 121 | 1.7% (0.2%-6.0%) |
| Ren *et al.* 2022^50^ | 126 | 0 | | 93 | 3.9 | 33 | | 496 | 6.7% (4.6%-9.3%) |
| Santos *et al.* 2022^18^ | 71 | 6 | | 44 | 2.1 | 15 | | 150 | 10.0% (5.6%-16.5%) |
| **Synthesis** | **226** | **11** | | **137** | **3.5** | **50** | | **793** | **6.3% (4.7%-8.3%)** |
|  | | | | | | | | | |
| **Pooled annual hemorrhage rate from birth to presentation** | | | | | | | | | |
| **Authors and year** | **N** | | **As** | | **Hemorrhages** | | **Person-age-years** | | **Annual hemorrhage rate (95% CI)** |
| Kharkar *et al.* 2007^27^ | 13 | | 0 | | 10 | | 543 | | 1.8% (0.9%-3.4%) |
| Bian *et al.* 2009^29^ | 16 | | 0 | | 19 | | 611 | | 3.1% (1.9%-4.9%) |
| Park *et al.* 2009^30^ | 14 | | 0 | | 8 | | 480 | | 1.7% (0.7%-3.3%) |
| Choi *et al.* 2011^2^ | 21 | | 0 | | 18 | | 826 | | 2.2% (1.3%-3.4%) |
| Badhiwala *et al.* 2014^53^ | 24 | | 0 | | 3 | | 968 | | 0.3% (0.1%-0.9%) |
| Zhang *et al.* 2021^43^ | 111 | | 0 | | 121 | | 4,506 | | 2.7% (2.2%-3.2%) |
| Zhang *et al.* 2021^44^ | 18 | | 0 | | 18 | | 233 | | 7.7% (4.6%-12.2%) |
| Cai *et al.* 2023^46^ | 29 | | 0 | | 27 | | 1,310 | | 2.1% (1.4%-3.0%) |
| **Synthesis** | **246** | | **0** | | **224** | | **9,477** | | **2.4% (2.1%-2.7%)** |
|  | | | | | | | | | |
| **Pooled annual hemorrhage rate from birth to surgery** | | | | | | | | | |
| **Authors and year** | **N** | | **As** | | **Hemorrhages** | | **Person-age-years** | | **Annual hemorrhage rate (95% CI)** |
| Sandalcioglu *et al.* 2003^25^ | 10 | | 0 | | 17 | | 399 | | 4.3% (2.5%-6.8%) |
| Aoyama *et al.* 2011^32^ | 12 | | 0 | | 28 | | 449 | | 6.2% (4.1%-9.0%) |
| Choi *et al.* 2011^2^ | 21 | | 0 | | 27 | | 885 | | 3.1% (2.0%-4.4%) |
| Zhang *et al.* 2021^44^ | 18 | | 0 | | 22 | | 248 | | 8.9% (5.6%-13.4%) |
| **Synthesis** | **61** | | **0** | | **94** | | **1,981** | | **4.7% (3.8%-5.8%)** |

Data are number, mean, or rate (%); in Zhang *et al.* 2016^15^ and Goyal *et al.* 2019^40^ only the patients who were (initially) managed conservatively were assessed, Santos *et al.* 2022^18^ reported more person-years of follow-up in their per-patient analysis of hemorrhage and fewer person-years of follow-up in their per-hemorrhage analysis without clarifying this ambiguity; Zhang *et al.* 2021^44^ was a study of pediatric cases and therefore their denominator of person-age-years was relatively low; As, asymptomatic; CI, confidence interval; FU, follow-up; Hx, history of hemorrhage at start of follow-up; N, number.

Supplemental Table 7. Neurological outcome postoperatively and at final follow-up for patients who underwent surgical treatment

| **Authors and year** | **N** | **Symptom duration (±SD)** | **IO NM** | **CR** | **Postoperative** | | **Neurological outcome postoperatively** | | | | **Postoperative follow-up (±SD)** | **Neurological outcome at final follow-up** | | | |
| --- | --- | --- | --- | --- | --- | --- | --- | --- | --- | --- | --- | --- | --- | --- | --- |
|  |  |  |  |  | **Complication** | **Hemorrhage** |  |  |  |  |  |  |  |  |  |
|  |  |  |  |  |  |  | **I** | **S** | **W** | **U** |  | **I** | **S** | **W** | **U** |
| Cosgrove *et al.* 1988^20^ | 5 | 46 (±54) |  | 3 |  |  |  |  |  |  | 70 (±81) | 1 | 3 | 1 | 0 |
| McCormick *et al.* 1988^21^ | 6 | 91^1^ (0-300)^2^ |  | 5 |  |  |  |  |  |  | 37 (4-84)^2^ | 4 | 2 | 0 | 0 |
| Ogilvy *et al.* 1992^9^ | 6 | 63 (±58) |  | 5 |  | 1 |  |  |  |  | 11 (±8) | 4 | 1 | 1 | 0 |
| Cantore *et al.* 1995^22^ | 6 | 55 (±39) |  | 6 |  |  |  |  |  |  | 12 (±5) | 2 | 3 | 1 | 0 |
| Spetzger *et al.* 1995^8^ | 9 | 42 |  | 9 |  |  | 1 | 3 | 5 | 0 | 14 | 8 | 1 | 0 | 0 |
| Padovani *et al.* 1997^23^ | 6 | 3 (±2) | 1 | 6 |  | 0 |  |  |  |  | 22 (±9) | 5 | 1 | 0 | 0 |
| Tu *et al.* 1999^24^ | 7 |  | 7 | 7 | 0 |  | 0 | 0 | 4 | 3 | (6-80)^2^ | 6 | 1 | 0 | 0 |
| Shan *et al.* 2002^51^ | 21 |  |  | 18 |  |  | 15 | 4 | 2 | 0 | 49 (10-108)^2^ | 7 | 2 | 1 | 11 |
| Sandalcioglu *et al.* 2003^25^ | 10 | 29 (±54) | 10 | 10 | 0 | 0 | 4 | 1 | 5 | 0 | 11 (±8) | 4 | 6 | 0 | 0 |
| Santoro *et al.* 2004^26^ | 10 | 29 (±48) |  | 10 |  |  | 0 | 8 | 2 | 0 | 68 (±34) | 9 | 1 | 0 | 0 |
| Jallo *et al.* 2006^11^ | 26 | 44 (±76) | 26 | 25 | 0 |  | 5 | 8 | 13 | 0 | 54 (±31) | 12 | 12 | 2 | 0 |
| Kharkar *et al.* 2007^27^ | 4 | 58 (±82) |  |  |  |  |  |  |  |  | 43 (±71) | 1 | 2 | 1 | 0 |
| Gu *et al.* 2008^28^ | 28 | 18 (3-144)^2^ |  |  |  |  |  |  |  |  | (3-72)^2^ | 16 | 8 | 4 | 0 |
| Labauge *et al.* 2008^52^ | 40 |  |  |  |  |  |  |  |  |  | 42 (12-288)^2^ | 20 | 6 | 11 | 3 |
| Bian *et al.* 2009^29^ | 16 | 34 (±76) | 16 | 16 | 0 |  |  |  |  |  | 23 (1-78)^2^ | 12 | 4 | 0 | 0 |
| Park *et al.* 2009^30^ | 14 | 32 (±32) |  | 14 |  |  | 11 | 0 | 3 | 0 | 55 (±45) | 7 | 4 | 3 | 0 |
| Deutsch 2010^31^ | 5 | 62 | 5 | 5 |  |  |  |  |  |  | 12 (12-12)^2^ | 5 | 0 | 0 | 0 |
| Steiger *et al.* 2010^10^ | 17 | 22^1^ (±35) | 17 | 17 |  |  |  |  |  |  | 30 (±10) | 11 | 3 | 3 | 0 |
| Aoyama *et al.* 2011^32^ | 12 | 40 (±57) | 2 |  |  | 3 |  |  |  |  | 76 (±69) | 9 | 3 | 0 | 0 |
| Choi *et al.* 2011^2^ | 21 | 34 (±68) | 21 | 20 | 0 | 1 | 7 | 11 | 3 | 0 | 33 (±23) | 10 | 9 | 2 | 0 |
| Maslehaty *et al.* 2011^33^ | 11 | 11^1^ (±17) | 11 | 9 |  | 1 | 4 | 3 | 4 | 0 | 19 (±15) | 10 | 1 | 0 | 0 |
| Wachter *et al.* 2012^34^ | 30 |  | Yes | 28 | 2^7^ | 2 | 3 | 10 | 17 | 0 | (2-31)^2^ | 15 | 12 | 3 | 0 |
| Badhiwala *et al.* 2014^53^ | 11 |  |  | 10 |  |  | 0 | 0 | 6 | 5 | 12 | 6 | 3 | 2 | 0 |
| Li *et al.* 2014^35^ | 21 | 29 (24-432)^2^ | 21 | 20 |  |  |  |  |  |  | 46 (8-60)^2^ | 13 | 6 | 2 | 0 |
| Reitz *et al.* 2015^7^ | 48 | 16^1^ (±20) | 48 | 48 | 3^7^ |  | 6 | 26 | 16 | 0 | 79 (±35) | 11 | 34 | 3 | 0 |
| Zhang *et al.* 2016^15^ | 58 | 24^1^ (±47) | 58 | 51 | 0 | 0 |  |  |  |  | 47 (±28) | 20 | 36 | 2 | 0 |
| Imagama *et al.* 2017^13^ | 41 | 20^1^ (±26) | 41 | 37 |  | 0 |  |  |  |  | 120 (24-288)^2^ |  |  |  |  |
| Sun *et al.* 2017^36^ | 10 |  | 10 | 10 | 1^7^ |  |  |  |  |  | 55 (±44) | 8 | 1 | 1 | 0 |
| Azad *et al.* 2018^37^ | 32 | 28 (±37) | 30 | 32 | 2^7^ |  |  |  |  |  | 50 (±52) | 6 | 19 | 1 | 6 |
| Ghobrial *et al.* 2018^38^ | 13 |  | 13 |  |  |  |  |  |  |  | 8 (±7) |  |  |  |  |
| Li *et al.* 2018^12^ | 83 | 5^3^ (2-13)^4^ | 83 | 81 | 0 |  | 18 | 57 | 8 | 0 | 57^3^ (31-102)^4^ | 19 | 39 | 5 | 20 |
| Velz *et al.* 2018^39^ | 21 | 13 (±23) | 17 | 18 | 3^7^ |  | 2 | 13 | 6 | 0 | 36 (±7) | 15 | 6 | 0 | 0 |
| Goyal *et al.* 2019^40^ | 21 | 27^1^ (±34) | Yes |  | 2^7^ |  |  |  |  |  |  | 10 | 15 | 7 | 0 |
| Nagoshi *et al.* 2019^41^ | 57 | 27 (±40) | 57 | 54 | 17^7^ | 0 |  |  |  |  | 53 (±31) |  |  |  |  |
| Ohnishi *et al.* 2020^42^ | 5 | 25 (3-60)^2^ | 5 | 5 | 0 | 0 |  |  |  |  | 52 (6-142)^2^ |  |  |  |  |
| Zhang *et al.* 2021^43^ | 111 | 20 (±35) | 111 | 96 | 3^7^ |  | 21 | 77 | 13 | 0 | 110 (±50) | 57 | 46 | 8 | 0 |
| Zhang *et al.* 2021^44^ | 16 | 13 (±25) | 16 | 12 | 2^7^ |  | 2 | 12 | 2 | 0 | 112 (±60) | 8 | 7 | 1 | 0 |
| Liao *et al.* 2022^17^ | 98 | 10 (±13) | 98 | 94 |  |  |  |  |  |  | 34 (6-60)^2^ | 34 | 41 | 6 | 17 |
| Niedermeyer *et al.* 2022^45^ | 17 |  | 17 | 17 |  | 0 |  |  |  |  | 12 (12-12)^2^ | 5 | 12 | 0 | 0 |
| Cai *et al.* 2023^46^ | 29 | 24^1^ (±42) | 29 | 29 |  | 0 |  |  |  |  | 58 (19-106)^2^ | 19 | 4 | 6 | 0 |
| Chen *et al.* 2023^47^ | 19 |  | 19 |  | 0 | 0 |  |  |  |  | 27 (±9) | 14 | 5 | 0 | 0 |
| Kurokawa *et al.* 2023^16^ | 160 | 33^5^ (2-35)^4^ |  | 142 | 7^7^ |  |  |  |  |  | 46 (0-142)^2^ | 60 | 82 | 18 | 0 |
| Liu *et al.* 2023^19^ | 268 | 23^1^ | 268 | 268 | 0 | 0 |  |  |  |  | 12 (12-12)^2^ | 167 | 50 | 51 | 0 |
| Srinivasan *et al.* 2023^6^ | 146 | 0^6^ (±0) |  | 129 | 5^7^ |  | 10 | 92 | 21 | 23 | 43 (±54) | 28 | 68 | 9 | 41 |
| Tian *et al.* 2023^14^ | 279 | 15 (±29) | 279 | 275 |  |  |  |  |  |  | 32 (±28) | 110 | 159 | 10 | 0 |
| Früh *et al.* 2025^54^ | 35 |  | 22 |  |  |  |  |  |  |  | 4^3^ (3-7)^4^ |  |  |  |  |
| **Synthesis** | **1,909** | **21 (±36)** | **98%** | **94%** | **4%** | **2%** | **109** | **325** | **130** | **31** | **43 (±38)** | **788** | **718** | **165** | **98** |

Data are number, mean time in months (±SD), number (%), or otherwise specified; the weighted mean duration of symptoms between onset and surgery was 21 months (n=1,623, 35 studies) with a pooled SD of ±36 months (n=1,014, 27 studies); intraoperative neuromonitoring (IONM) was used in 98% (1,358/1,392, 30 studies); complete resection was achieved in 94% (1,641/1,737, 38 studies); postoperative complications occurred in 4% (47/1,165, 21 studies); postoperative (re)hemorrhage occurred in 2% (9/590, all from a residual lesion, 15 studies); the weighted mean postoperative follow-up was 43 months (n=1,638, 40 studies) with a pooled SD of ±38 months (n=922, 25 studies); Goyal *et al.* 2019^40^ provided the neurological outcome only according to the eventually applied strategy (n=32); CR, complete resection; I, improved; IONM, intraoperative neuromonitoring; N, number; S, stable; SD, standard deviation; U, unknown; W, worsened.

^1^Duration of symptoms between onset and admission

^2^Range

^3^Median

^4^Interquartile range (IQR)

^5^In addition, the median time from presentation to surgery was 32 days

^6^Duration of nonacute symptoms between onset and unknown

^7^Wachter *et al.* 2012^34^: n=1 neuropathic pain and n=1 contralateral paresis; Reitz *et al.* 2015^7^: n=1 recurrent lesion, n=1 acute epidural hematoma, and n=1 kyphosis; Sun *et al.* 2017^36^: n=1 left hemiparesis; Azad *et al.* 2018^37^: n=2 cerebrospinal fluid leak; Velz *et al.* 2018^39^: early n=2 wound infection, and late n=1 kyphosis, n=1 hypertrophic scarring, and n=1 neuropathic pain; Goyal *et al.* 2019^40^: n=1 spinal cord tethering and n=1 posterior column dysfunction; Nagoshi *et al.* 2019^41^: n=17 of whom n=16 neurological deficits, n=1 infection, n=1 depression, and n=1 kyphosis; Zhang *et al.* 2021^43^: n=3 recurrent lesion; Zhang *et al.* 2021^44^: n=1 recurrent lesion and n=1 scoliosis; Kurokawa *et al.* 2023^16^: n=7 recurrent lesion; Srinivasan *et al.* 2023^6^: unknown

Supplemental Table 8. Standardized scoring tool outcomes for patients who underwent surgical treatment

| **Authors and year** | **McCormick scale** | | | | | | | | | | | | | | | | | | | | | | | |
| --- | --- | --- | --- | --- | --- | --- | --- | --- | --- | --- | --- | --- | --- | --- | --- | --- | --- | --- | --- | --- | --- | --- | --- | --- |
|  | **Preoperative** | | | | | | | | **Postoperative** | | | | | | | | **Final follow-up** | | | | | | | |
|  | **1** | | **2** | | **3** | | **4** | | **1** | | **2** | | **3** | | **4** | | **1** | | **2** | | **3** | | **4** | |
| Labauge *et al.* 2008^52^ | 13 | | 11 | | 10 | | 3 | |  | |  | |  | |  | | 16 | | 6 | | 12 | | 3 | |
| Aoyama *et al.* 2011^32^ | 2 | | 4 | | 1 | | 5 | |  | |  | |  | |  | | 7 | | 2 | | 3 | | 0 | |
| Maslehaty *et al.* 2011^33^ | 4 | | 4 | | 2 | | 1 | | 4 | | 2 | | 5 | | 0 | | 8 | | 2 | | 0 | | 0 | |
| Sun *et al.* 2017^36^ | 3 | | 5 | | 2 | | 0 | | 4 | | 5 | | 0 | | 1 | | 8 | | 2 | | 0 | | 0 | |
| Li *et al.* 2018^12^ | 36 | | 26 | | 11 | | 10 | | 41 | | 24 | | 13 | | 5 | | 31 | | 19 | | 12 | | 1 | |
| Niedermeyer *et al.* 2022^45^ | 5 | | 8 | | 4 | | 0 | | 3 | | 10 | | 3 | | 1 | | 9 | | 5 | | 3 | | 0 | |
| Früh *et al.* 2025^54^ | 17 | | 13 | | 5 | | 0 | | 18 | | 12 | | 5 | | 0 | | 22 | | 8 | | 5 | | 0 | |
| **Synthesis** | **80** | | **71** | | **35** | | **19** | | **70** | | **53** | | **26** | | **7** | | **101** | | **44** | | **35** | | **4** | |
|  | | | | | | | | | | | | | | | | | | | | | | | | |
| **Authors and year** | **Modified McCormick scale** | | | | | | | | | | | | | | | | | | | | | | | |
|  | **Preoperative** | | | | | | | | **Postoperative** | | | | | | | | **Final follow-up** | | | | | | | |
|  | **1** | **2** | | **3** | | **4** | | **5** | **1** | **2** | | **3** | | **4** | | **5** | **1** | **2** | | **3** | | **4** | | **5** |
| Kharkar *et al.* 2007^27^ | 0 | 2 | | 0 | | 1 | | 1 |  |  | |  | |  | |  | 1 | 0 | | 1 | | 1 | | 1 |
| Choi *et al.* 2011^2^ | 6 | 7 | | 5 | | 2 | | 1 | 8 | 7 | | 5 | | 1 | | 0 | 9 | 10 | | 2 | | 0 | | 0 |
| Zhang *et al.* 2016^15^ | 0 | 18 | | 24 | | 6 | | 10 |  |  | |  | |  | |  | 23 | 12 | | 12 | | 5 | | 6 |
| Imagama *et al.* 2017^13^ | 19 | 9 | | 3 | | 3 | | 7 |  |  | |  | |  | |  | 35 | | | 6 | | | | |
| Ghobrial *et al.* 2018^38^ | Median 2 (IQR 1.0) | | | | | | | | Median 2 (IQR 1.0) | | | | | | | | Median 2 (IQR 1.5) | | | | | | | |
| Nagoshi *et al.* 2019^41^ | 22 | 14 | | 15 | | 5 | | 1 | 23 | 17 | | 14 | | 0 | | 3 | 25 | 13 | | 16 | | 0 | | 3 |
| Ohnishi *et al.* 2020^42^ | 0 | 0 | | 1 | | 2 | | 2 |  |  | |  | |  | |  | 0 | 3 | | 1 | | 0 | | 1 |
| Zhang *et al.* 2021^43^ | 7 | 36 | | 38 | | 14 | | 16 | 19 | 30 | | 27 | | 19 | | 16 | 37 | 30 | | 20 | | 9 | | 15 |
| Zhang *et al.* 2021^44^ | 0 | 7 | | 3 | | 3 | | 3 | 0 | 7 | | 3 | | 2 | | 4 | 3 | 7 | | 4 | | 0 | | 2 |
| Cai *et al.* 2023^46^ | 4 | 10 | | 8 | | 3 | | 4 |  |  | |  | |  | |  | 14 | 3 | | 3 | | 6 | | 3 |
| Kurokawa *et al.* 2023^16^ | 14 | 67 | | 39 | | 30 | | 10 |  |  | |  | |  | |  | 33 | 71 | | 32 | | 17 | | 7 |
| Tian *et al.* 2023^14^ | 35 | 153 | | 28 | | 27 | | 36 |  |  | |  | |  | |  | Mean 2,04 (SD ±0,99) | | | | | | | |
| **Synthesis** | **107** | **323** | | **164** | | **96** | | **91** | **50** | **61** | | **49** | | **22** | | **23** | **145** | **149** | | **91** | | **38** | | **38** |
| **Synthesis including Imagama *et al.* 2017^13^** |  | | | | | | | | | | | | | | | | **329** | | | **173** | | | | |
|  | | | | | | | | | | | | | | | | | | | | | | | | |
| **Authors and year** | **Frankel scale** | | | | | | | | | | | | | | | | | | | | | | | |
|  | **Preoperative** | | | | | | | | **Postoperative** | | | | | | | | **Final follow-up** | | | | | | | |
|  | **A** | **B** | | **C** | | **D** | | **E** | **A** | **B** | | **C** | | **D** | | **E** | **A** | **B** | | **C** | | **D** | | **E** |
| Sandalcioglu *et al.* 2003^25^ | 1 | 1 | | 2 | | 6 | | 0 |  |  | |  | |  | |  | 0 | 0 | | 0 | | 10 | | 0 |
| Bian *et al.* 2009^29^ | 0 | 0 | | 8 | | 8 | | 0 |  |  | |  | |  | |  | 0 | 0 | | 2 | | 8 | | 6 |
| Steiger *et al.* 2010^10^ | 0 | 0 | | 6 | | 8 | | 3 |  |  | |  | |  | |  | 0 | 0 | | 2 | | 9 | | 6 |
| Maslehaty *et al.* 2011^33^ | 1 | 0 | | 2 | | 4 | | 4 | 0 | 1 | | 5 | | 1 | | 4 | 0 | 0 | | 0 | | 3 | | 7 |
| Azad *et al.* 2018^37^ | 0 | 3 | | 3 | | 14 | | 12 | 0 | 0 | | 5 | | 14 | | 12 | 0 | 0 | | 0 | | 14 | | 12 |
| Velz *et al.* 2018^39^ | 1 | 0 | | 2 | | 15 | | 3 | 0 | 1 | | 2 | | 17 | | 1 | 0 | 0 | | 1 | | 12 | | 8 |
| **Synthesis** | **3** | **4** | | **23** | | **55** | | **22** | **0** | **2** | | **12** | | **32** | | **17** | **0** | **0** | | **5** | | **56** | | **39** |
|  | | | | | | | | | | | | | | | | | | | | | | | | |
| **Authors and year** | **Aminoff-Logue Disability Scale** | | | | | | | | | | | | | | | | | | | | | | | |
|  | **Preoperative** | | | | | | | | **Postoperative** | | | | | | | | **Final follow-up** | | | | | | | |
|  | **I** | | **II** | | **III** | | **IV** | | **I** | | **II** | | **III** | | **IV** | | **I** | | **II** | | **III** | | **IV** | |
| Azad *et al.* 2018^37^ | 21 | | 5 | | 1 | | 3 | | 12 | | 9 | | 7 | | 2 | | 16 | | 6 | | 2 | | 0 | |
| Zhang *et al.* 2021^43^ | 36 | | 41 | | 17 | | 17 | | 38 | | 41 | | 15 | | 17 | | 54 | | 33 | | 12 | | 12 | |
| **Synthesis** | **57** | | **46** | | **18** | | **20** | | **50** | | **50** | | **22** | | **19** | | **70** | | **39** | | **14** | | **12** | |
|  | | | | | | | | | | | | | | | | | | | | | | | | |
| **Authors and year** | **ASIA Impairment Scale** | | | | | | | | | | | | | | | | | | | | | | | |
|  | **Preoperative** | | | | | | | | **Postoperative** | | | | | | | | **Final follow-up** | | | | | | | |
|  | **A** | **B** | | **C** | | **D** | | **E** | **A** | **B** | | **C** | | **D** | | **E** | **A** | **B** | | **C** | | **D** | | **E** |
| Reitz *et al.* 2015^7^ | 3 | 0 | | 10 | | 21 | | 14 | 1 | 1 | | 18 | | 22 | | 6 | 1 | 1 | | 6 | | 23 | | 17 |
| Nagoshi *et al.* 2019^41^ | 1 | 0 | | 7 | | 42 | | 7 | 3 | 0 | | 6 | | 37 | | 11 | 3 | 0 | | 5 | | 36 | | 13 |
| Liao *et al.* 2022^17^ | 2 | 2 | | 12 | | 53 | | 29 | 0 | 4 | | 27 | | 51 | | 16 | 0 | 0 | | 8 | | 40 | | 33 |
| Liu *et al.* 2023^19^ | 7 | 15 | | 36 | | 154 | | 56 |  |  | |  | |  | |  |  |  | |  | |  | |  |
| **Synthesis** | **13** | **17** | | **65** | | **270** | | **106** | **4** | **5** | | **51** | | **110** | | **33** | **4** | **1** | | **19** | | **99** | | **63** |

Data are number or otherwise specified; ASIA, American Spinal Injury Association; IQR, interquartile range; SD, standard deviation.


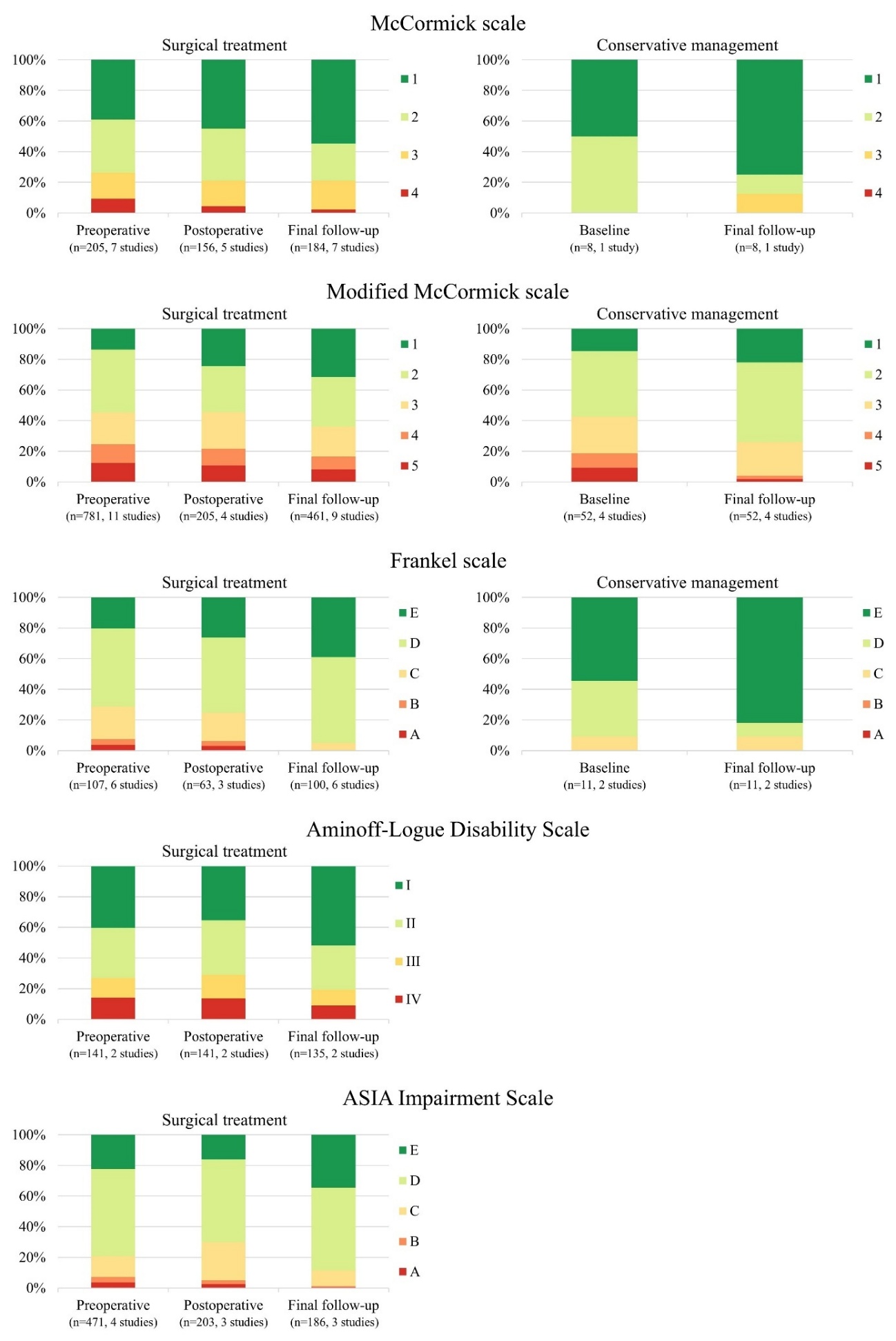


Supplemental Figure 3. Distributions across the five standardized outcome tools stratified by treatment strategy; ASIA, American Spinal Injury Association

Supplemental Table 9. Neurological outcome at final follow-up for patients underwent conservative management

| **Authors and year** | **N** | **Follow-up** | **Neurological outcome at final follow-up** | | | |
| --- | --- | --- | --- | --- | --- | --- |
|  |  |  | **I** | **S** | **W** | **U** |
| Shan *et al.* 2002^51^ | 2 | ? |  |  |  |  |
| Kharkar *et al.* 2007^27^ | 10 | 80 (±56) | 1 | 8 | 1 | 0 |
| Labauge *et al.* 2008^52^ | 13 | 43 (±23) | 1 | 5 | 1 | 6 |
| Steiger *et al.* 2010^10^ | 3 | 56 (±49) | 2 | 1 | 0 | 0 |
| Hegde *et al.* 2012^48^ | 6 | 56 (±41) | 3 | 0 | 3 | 0 |
| Kim *et al.* 2013^49^ | 24 | 60 (±19) |  |  |  |  |
| Badhiwala *et al.* 2014^53^ | 13 | ? | 2 | 10 | 1 | 0 |
| Zhang *et al.* 2016^15^ | 27 | 34 (±21) | 4 | 19 | 4 | 0 |
| Velz *et al.* 2018^39^ | 8 | 36 (±7) | 2 | 6 | 0 | 0 |
| Goyal *et al.* 2019^40^ | 64 | 63 | 7 | 43 | 2 | 1 |
| Nagoshi *et al.* 2019^41^ | 9 | 49 (±28) |  |  |  |  |
| Ohnishi *et al.* 2020^42^ | 13 | 64 (24-151)^1^ |  |  |  |  |
| Zhang *et al.* 2021^44^ | 2 | 217 (±28) | 1 | 1 | 0 | 0 |
| Ren *et al.* 2022^50^ | 126 | 47 (6-244)^1^ | 83 | | 43 | 0 |
| Santos *et al.* 2022^18^ | 71 | 25 (±42) |  |  |  |  |
| Chen *et al.* 2023^47^ | 11 | 27 (±9) | 1 | 8 | 2 | 0 |
| Früh *et al.* 2025^54^ | 17 | 4^2^ (3-7)^3^ |  |  |  |  |
| **Synthesis** | **419** | **47 (±34)** | **24** | **101** | **14** | **7** |
| **Synthesis including Ren *et al.* 2022^50^** | | | **208** | | **57** | **7** |

Data are number, mean time in months (±SD), or otherwise specified; the weighted mean follow-up was 47 months (n=387, 14 studies) with a pooled SD of ±34 months (n=184, 11 studies); Goyal *et al.* 2019^40^ did not provide the neurological outcome for the patients initially assigned to conservative management (n=64) but only for those eventually managed conservatively (n=53); I, improved; N, number; S, stable; SD, standard deviation; U, unknown; W, worsened.

^1^Range

^2^Median

^3^Interquartile range (IQR)

Supplemental Table 10. Standardized scoring tool outcomes for patients who underwent conservative management

| **Authors and year** | **McCormick scale** | | | | | | | | | | | | | | | |
| --- | --- | --- | --- | --- | --- | --- | --- | --- | --- | --- | --- | --- | --- | --- | --- | --- |
|  | **Baseline** | | | | | | | | **Final follow-up** | | | | | | | |
|  | **1** | | **2** | | **3** | | **4** | | **1** | | **2** | | **3** | | **4** | |
| Früh *et al.* 2025^54^ | 4 | | 4 | | 0 | | 0 | | 6 | | 1 | | 1 | | 0 | |
|  | | | | | | | | | | | | | | | | |
| **Authors and year** | **Modified McCormick scale** | | | | | | | | | | | | | | | |
|  | **Baseline** | | | | | | | | **Final follow-up** | | | | | | | |
|  | **1** | **2** | | **3** | | **4** | | **5** | **1** | **2** | | **3** | | **4** | | **5** |
| Kharkar *et al.* 2007^27^ | 3 | 4 | | 2 | | 0 | | 1 | 3 | 4 | | 2 | | 1 | | 0 |
| Zhang *et al.* 2016^15^ | 5 | 13 | | 7 | | 2 | | 0 | 5 | 15 | | 6 | | 0 | | 1 |
| Ohnishi *et al.* 2020^42^ | 0 | 6 | | 3 | | 3 | | 1 | 2 | 7 | | 3 | | 0 | | 1 |
| Zhang *et al.* 2021^44^ | 0 | 0 | | 1 | | 0 | | 1 | 1 | 0 | | 0 | | 0 | | 1 |
| Ren *et al.* 2022^50^ | 106 | | | 20 | | | | |  |  | |  | |  | |  |
| **Synthesis** | **8** | **23** | | **13** | | **5** | | **3** | **11** | **26** | | **11** | | **1** | | **3** |
| **Synthesis including Ren *et al.* 2022^50^** | **137** | | | **41** | | | | |  | | | | | | | |
|  | | | | | | | | | | | | | | | | |
| **Authors and year** | **Frankel scale** | | | | | | | | | | | | | | | |
|  | **Baseline** | | | | | | | | **Final follow-up** | | | | | | | |
|  | **A** | **B** | | **C** | | **D** | | **E** | **A** | **B** | | **C** | | **D** | | **E** |
| Steiger *et al.* 2010^10^ | 0 | 0 | | 0 | | 2 | | 1 | 0 | 0 | | 0 | | 0 | | 3 |
| Velz *et al.* 2018^39^ | 0 | 0 | | 1 | | 2 | | 5 | 0 | 0 | | 1 | | 1 | | 6 |
| **Synthesis** | **0** | **0** | | **1** | | **4** | | **6** | **0** | **0** | | **1** | | **1** | | **9** |

Data are number.

Supplemental Table 11. Comparative cohort analysis of the neurological outcome after either treatment strategy

| **Authors and year** | **N** | **Neurological outcome after surgical treatment** | | | | | | | |
| --- | --- | --- | --- | --- | --- | --- | --- | --- | --- |
|  |  | **Postoperatively** | | | | **At final follow-up** | | | |
|  |  | **I** | **S** | **W** | **U** | **I** | **S** | **W** | **U** |
| Shan *et al.* 2002^51^ | 21 | 15 | 4 | 2 | 0 | 7 | 2 | 1 | 11 |
| Kharkar *et al.* 2007^27^ | 4 |  |  |  |  | 1 | 2 | 1 | 0 |
| Labauge *et al.* 2008^52^ | 40 |  |  |  |  | 20 | 6 | 11 | 3 |
| Steiger *et al.* 2010^10^ | 17 |  |  |  |  | 11 | 3 | 3 | 0 |
| Badhiwala *et al.* 2014^53^ | 11 | 0 | 0 | 6 | 5 | 6 | 3 | 2 | 0 |
| Zhang *et al.* 2016^15^ | 58 |  |  |  |  | 20 | 36 | 2 | 0 |
| Velz *et al.* 2018^39^ | 21 | 2 | 13 | 6 | 0 | 15 | 6 | 0 | 0 |
| Goyal *et al.* 2019^40^ | 21 |  |  |  |  | 10 | 15 | 7 | 0 |
| Nagoshi *et al.* 2019^41^ | 57 |  |  |  |  |  |  |  |  |
| Ohnishi *et al.* 2020^42^ | 5 |  |  |  |  |  |  |  |  |
| Zhang *et al.* 2021^44^ | 16 | 2 | 12 | 2 | 0 | 8 | 7 | 1 | 0 |
| Chen *et al.* 2023^47^ | 19 |  |  |  |  | 14 | 5 | 0 | 0 |
| Früh *et al.* 2025^54^ | 35 |  |  |  |  |  |  |  |  |
| **Synthesis** | **325** | **19** | **29** | **16** | **5** | **112** | **85** | **28** | **14** |
|  | | | | | | | | | |
| **Authors and year** | **N** | **Neurological outcome after conservative management at final follow-up** | | | | | | | |
|  |  | **I** | | **S** | | **W** | | **U** | |
| Shan *et al.* 2002^51^ | 2 |  | |  | |  | |  | |
| Kharkar *et al.* 2007^27^ | 10 | 1 | | 8 | | 1 | | 0 | |
| Labauge *et al.* 2008^52^ | 13 | 1 | | 5 | | 1 | | 6 | |
| Steiger *et al.* 2010^10^ | 3 | 2 | | 1 | | 0 | | 0 | |
| Badhiwala *et al.* 2014^53^ | 13 | 2 | | 10 | | 1 | | 0 | |
| Zhang *et al.* 2016^15^ | 27 | 4 | | 19 | | 4 | | 0 | |
| Velz *et al.* 2018^39^ | 8 | 2 | | 6 | | 0 | | 0 | |
| Goyal *et al.* 2019^40^ | 64 | 7 | | 43 | | 2 | | 1 | |
| Nagoshi *et al.* 2019^41^ | 9 |  | |  | |  | |  | |
| Ohnishi *et al.* 2020^42^ | 13 |  | |  | |  | |  | |
| Zhang *et al.* 2021^44^ | 2 | 1 | | 1 | | 0 | | 0 | |
| Chen *et al.* 2023^47^ | 11 | 1 | | 8 | | 2 | | 0 | |
| Früh *et al.* 2025^54^ | 17 |  | |  | |  | |  | |
| **Synthesis** | **192** | **21** | | **101** | | **11** | | **7** | |

Data are number; Goyal *et al.* 2019^40^ did not provide the neurological outcome according to the initial treatment assignment (n=21 surgical treatment, n=64 conservative management) but only according to the eventually applied strategy (n=32 surgical treatment, n=53 conservative management); I, improved; N, number; S, stable; U, unknown; W, worsened.

Supplemental Table 12. Comparative cohort analysis of independent ambulation after either treatment strategy

| **Authors and year** | **Ambulation of surgically treated patients** | | | | | | | |
| --- | --- | --- | --- | --- | --- | --- | --- | --- |
|  | **Preoperative** | | | **Postoperative** | | **Final follow-up** | | |
|  | **Indep** | **Dep** | | **Indep** | **Dep** | **Indep** | | **Dep** |
| Shan *et al.* 2002^51^ |  |  | |  |  |  | |  |
| Kharkar *et al.* 2007^27^ | 2 | 2 | |  |  | 1 | | 3 |
| Labauge *et al.* 2008^52^ | 24 | 13 | |  |  | 22 | | 15 |
| Steiger *et al.* 2010^10^ | 11 | 6 | |  |  | 15 | | 2 |
| Badhiwala *et al.* 2014^53^ |  |  | |  |  |  | |  |
| Zhang *et al.* 2016^15^ | 18 | 40 | |  |  | 35 | | 23 |
| Velz *et al.* 2018^39^ | 18 | 3 | | 18 | 3 | 20 | | 1 |
| Goyal *et al.* 2019^40^ |  |  | |  |  |  | |  |
| Nagoshi *et al.* 2019^41^ | 36 | 21 | | 40 | 17 | 38 | | 19 |
| Ohnishi *et al.* 2020^42^ | 0 | 5 | |  |  | 3 | | 2 |
| Zhang *et al.* 2021^44^ | 7 | 9 | | 7 | 9 | 10 | | 6 |
| Chen *et al.* 2023^47^ |  |  | |  |  |  | |  |
| Früh *et al.* 2025^54^ | 30 | 5 | | 30 | 5 | 30 | | 5 |
| **Synthesis** | **146** | **104** | | **95** | **34** | **174** | | **76** |
|  | | | | | | | | |
| **Authors and year** | **Ambulation of conservatively managed patients** | | | | | | | |
|  | **Baseline** | | | | **Final follow-up** | | | |
|  | **Independent** | | **Dependent** | | **Independent** | | **Dependent** | |
| Shan *et al.* 2002^51^ |  | |  | |  | |  | |
| Kharkar *et al.* 2007^27^ | 7 | | 3 | | 7 | | 3 | |
| Labauge *et al.* 2008^52^ |  | |  | |  | |  | |
| Steiger *et al.* 2010^10^ | 3 | | 0 | | 3 | | 0 | |
| Badhiwala *et al.* 2014^53^ |  | |  | |  | |  | |
| Zhang *et al.* 2016^15^ | 18 | | 9 | | 20 | | 7 | |
| Velz *et al.* 2018^39^ | 7 | | 1 | | 7 | | 1 | |
| Goyal *et al.* 2019^40^ |  | |  | |  | |  | |
| Nagoshi *et al.* 2019^41^ |  | |  | |  | |  | |
| Ohnishi *et al.* 2020^42^ | 6 | | 7 | | 9 | | 4 | |
| Zhang *et al.* 2021^44^ | 0 | | 2 | | 1 | | 1 | |
| Chen *et al.* 2023^47^ |  | |  | |  | |  | |
| Früh *et al.* 2025^54^ | 8 | | 0 | | 7 | | 1 | |
| **Synthesis** | **49** | | **22** | | **54** | | **17** | |

Data are number; Dep, dependent; Indep, independent.

**References**

1. McCormick PC, Torres R, Post KD, Stein BM. Intramedullary ependymoma of the spinal cord. *J Neurosurg*. Apr 1990;72(4):523-32. doi:10.3171/jns.1990.72.4.0523

2. Choi GH, Kim KN, Lee S, et al. The clinical features and surgical outcomes of patients with intramedullary spinal cord cavernous malformations. *Acta Neurochirurgica*. Aug 2011;153(8):1677-1685. doi:10.1007/s00701-011-1016-3

3. Frankel HL, Hancock DO, Hyslop G, et al. The value of postural reduction in the initial management of closed injuries of the spine with paraplegia and tetraplegia. *Paraplegia*. Nov 1969;7(3):179-92. doi:10.1038/sc.1969.30

4. Aminoff MJ, Logue V. The prognosis of patients with spinal vascular malformations. *Brain*. 1974;97:211-218.

5. Maynard FM, Jr., Bracken MB, Creasey G, et al. International Standards for Neurological and Functional Classification of Spinal Cord Injury. American Spinal Injury Association. *Spinal Cord*. May 1997;35(5):266-74. doi:10.1038/sj.sc.3100432

6. Srinivasan VM, Karahalios K, Shlobin NA, et al. Residual and Recurrent Spinal Cord Cavernous Malformations: Outcomes and Techniques to Optimize Resection and a Systematic Review of the Literature. *Operative Neurosurgery*. Jan 2023;24(1):44-54. doi:10.1227/ons.0000000000000456

7. Reitz M, Burkhardt T, Vettorazzi E, et al. Intramedullary spinal cavernoma: clinical presentation, microsurgical approach, and long-term outcome in a cohort of 48 patients. *Neurosurgical Focus*. Aug 2015;39(2):E19. doi:10.3171/2015.5.focus15153

8. Spetzger U, Gilsbach JM, Bertalanffy H. Cavernous angiomas of the spinal cord: clinical presentation, surgical strategy, and postoperative results. *Acta Neurochirurgica*. 1995;134(3):200-206. doi:10.1007/bf01417690

9. Ogilvy CS, Louis DN, Ojemann RG. Intramedullary cavernous angiomas of the spinal cord: clinical presentation, pathological features, and surgical management. *Neurosurgery*. Aug 1992;31(2):219-230. doi:10.1227/00006123-199208000-00007

10. Steiger HJ, Turowski B, Hänggi D. Prognostic factors for the outcome of surgical and conservative treatment of symptomatic spinal cord cavernous malformations: a review of a series of 20 patients. *Neurosurgical Focus*. Sep 2010;29(3):E13. doi:10.3171/2010.6.focus10123

11. Jallo GI, Freed D, Zareck M, Epstein F, Kothbauer KF. Clinical presentation and optimal management for intramedullary cavernous malformations. *Neurosurgical focus*. 2006;21:e10.

12. Li J, Chen G, Gu S, et al. Surgical Outcomes of Spinal Cord Intramedullary Cavernous Malformation: A Retrospective Study of 83 Patients in a Single Center over a 12-Year Period. *World Neurosurgery*. Oct 2018;118:e105-e114. doi:10.1016/j.wneu.2018.06.134

13. Imagama S, Ito Z, Ando K, et al. Optimal Timing of Surgery for Intramedullary Cavernous Hemangioma of the Spinal Cord in Relation to Preoperative Motor Paresis, Disease Duration, and Tumor Volume and Location. *Global Spine Journal*. May 2017;7(3):246-253. doi:10.1177/2192568217707938

14. Tian A, Cui Z, Ren J, et al. Surgical timing and long-term outcomes in patients with severe haemorrhagic spinal cord cavernous malformations. *Stroke and Vascular Neurology*. 2023:23002745. doi:10.1136/svn-2023-002745

15. Zhang L, Yang W, Jia W, et al. Comparison of outcome between surgical and conservative management of symptomatic spinal cord cavernous malformations. *Clinical Neurosurgery*. 2016;78:552-561. doi:10.1227/neu.0000000000001075

16. Kurokawa R, Endo T, Takami T. Acceptance of Early Surgery for Treatment of Spinal Cord Cavernous Malformation in Contemporary Japan. *Neurospine*. Jun 2023;20:587-594. doi:10.14245/ns.2346134.067

17. Liao D, Wang R, Shan B, Chen H. Surgical outcomes of spinal cavernous malformations: A retrospective study of 98 patients. *Front*. 2022;9:1075276. doi:10.3389/fsurg.2022.1075276

18. Santos AN, Rauschenbach L, Darkwah Oppong M, et al. Natural course of untreated spinal cord cavernous malformations: a follow-up study within the initial 5 years after diagnosis. *J Neurosurg Spine*. Jun 2022;36(6):1030-1034. doi:10.3171/2021.9.spine211052

19. Liu T, Wang L, Zhang S, et al. Prediction of outcomes for symptomatic spinal cavernous malformation surgery: a multicenter prospective clinical study. *European Spine Journal*. Apr 2023;32:1326-1333. doi:10.1007/s00586-023-07585-9

20. Cosgrove GR, Bertrand G, Fontaine S, Robitaille Y, Melanson D. Cavernous angiomas of the spinal cord. *J Neurosurg*. Jan 1988;68(1):31-6. doi:10.3171/jns.1988.68.1.0031

21. McCormick PC, Michelsen WJ, Post KD, et al. Cavernous malformations of the spinal cord. *Neurosurgery*. 1988;23:459-463. doi:10.1227/00006123-198810000-00009

22. Cantore G, Delfini R, Cervoni L, Innocenzi G, Orlando ER. Intramedullary cavernous angiomas of the spinal cord: Report of six cases. *Surgical Neurology*. 1995;43:448-452. doi:10.1016/0090-3019(95)80088-x

23. Padovani R, Acciarri N, Giulioni M, Pantieri R, Foschini MP. Cavernous angiomas of the spinal district: Surgical treatment of 11 patients. *European Spine Journal*. 1997;6:298-303. doi:10.1007/bf01142674

24. Tu YK, Liu HM, Chen SJ, Lin SM. Intramedullary cavernous haemangiomas: clinical features, imaging diagnosis, surgical resection and outcome. *J Clin Neurosci*. May 1999;6(3):212-216. doi:10.1054/jocn.1997.0026

25. Sandalcioglu IE, Wiedemayer H, Gasser T, Asgari S, Engelhorn T, Stolke D. Intramedullary spinal cord cavernous malformations: clinical features and risk of hemorrhage. *Neurosurgical Review*. Oct 2003;26(4):253-256. doi:10.1007/s10143-003-0260-2

26. Santoro A, Piccirilli M, Frati A, et al. Intramedullary spinal cord cavernous malformations: report of ten new cases. *Neurosurgical Review*. Apr 2004;27(2):93-98. doi:10.1007/s10143-003-0302-9

27. Kharkar S, Shuck J, Conway J, Rigamonti D. The natural history of conservatively managed symptomatic intramedullary spinal cord cavernomas. *Neurosurgery*. May 2007;60(5):865-871. doi:10.1227/01.neu.0000255437.36742.15

28. Gu XC, Yang YM, Yuan QG, Sha C, Jiang HZ. Microsurgical treatment of spinal cavernous malformation. *National Medical Journal of China*. Jun 2008;88:1621-1623.

29. Bian LG, Bertalanffy H, Sun QF, Shen JK. Intramedullary cavernous malformations: Clinical features and surgical technique via hemilaminectomy. *Clinical Neurology and Neurosurgery*. Jul 2009;111(6):511-517. doi:10.1016/j.clineuro.2009.02.003

30. Park SB, Jahng TA, Chung CK. The clinical outcomes after complete surgical resection of intramedullary cavernous angiomas: changes in motor and sensory symptoms. *Spinal Cord*. Feb 2009;47(2):128-133. doi:10.1038/sc.2008.89

31. Deutsch H. Pain outcomes after surgery in patients with intramedullary spinal cord cavernous malformations. *Neurosurgical Focus*. Sep 2010;29(3):E15. doi:10.3171/2010.6.focus10108

32. Aoyama T, Hida K, Houkin K. Intramedullary Cavernous Angiomas of the Spinal Cord: Clinical Characteristics of 13 Lesions. *Neurologia Medico-Chirurgica*. Aug 2011;51(8):561-566. doi:10.2176/nmc.51.561

33. Maslehaty H, Barth H, Petridis AK, Doukas A, Mehdorn HM. Symptomatic spinal cavernous malformations: indication for microsurgical treatment and outcome. *European Spine Journal*. Oct 2011;20(10):1765-1770. doi:10.1007/s00586-011-1898-z

34. Wachter D, Psychogios M, Gilsbach JM, Rohde V. Spinal Cord Cavernoma-Operative Strategy and Results in 30 Patients. *J Neurol Surg Part A*. May 2012;73(3):125-131. doi:10.1055/s-0032-1304811

35. Li Z, Li J, Qi L, Yang J, Xu Y, Wang G. Clinical features and microsurgical treatment of spinal intramedullary cavernoma. *National Medical Journal of China*. May 2014;94:1459-1461. doi:10.3760/cma.j.issn.0376-2491.2014.19.007

36. Sun I, Necmettin Pamir M. Spinal cavernomas: Outcome of surgically treated 10 patients. *Frontiers in Neurology*. Dec 2017;8:672. doi:10.3389/fneur.2017.00672

37. Azad TD, Veeravagu A, Li A, Zhang M, Madhugiri V, Steinberg GK. Long-Term Effectiveness of Gross-Total Resection for Symptomatic Spinal Cord Cavernous Malformations. *Neurosurgery*. Dec 2018;83(6):1201-1208. doi:10.1093/neuros/nyx610

38. Ghobrial GM, Liounakos J, Starke RM, Levi AD. Surgical Treatment of Vascular Intramedullary Spinal Cord Lesions. *Cureus J Med Sci*. Aug 2018;10(8):e3154. doi:10.7759/cureus.3154

39. Velz J, Bozinov O, Sarnthein J, Regli L, Bellut D. The current management of spinal cord cavernoma. *Journal of Neurosurgical Sciences*. Aug 2018;62(4):383-396. doi:10.23736/s0390-5616.18.04305-9

40. Goyal A, Rinaldo L, Alkhataybeh R, et al. Clinical presentation, natural history and outcomes of intramedullary spinal cord cavernous malformations. *Journal of Neurology Neurosurgery and Psychiatry*. Jun 2019;90(6):695-703. doi:10.1136/jnnp-2018-319553

41. Nagoshi N, Tsuji O, Nakashima D, et al. Clinical outcomes and prognostic factors for cavernous hemangiomas of the spinal cord: a retrospective cohort study. *J Neurosurg-Spine*. Aug 2019;31(2):271-278. doi:10.3171/2019.1.spine18854

42. Ohnishi YI, Nakajima N, Takenaka T, et al. Conservative and Surgical Management of Spinal Cord Cavernous Malformations. *World Neurosurgery*. Jan 2020;5:100066. doi:10.1016/j.wnsx.2019.100066

43. Zhang L, Yu X, Qiao G, Shang A. Long-term surgical outcomes and prognostic factors of adult symptomatic spinal cord cavernous malformations. *Journal of Clinical Neuroscience*. Aug 2021;90:171-177. doi:10.1016/j.jocn.2021.05.034

44. Zhang L, Qiao G, Yang W, Shang A, Yu X. Clinical features and long-term outcomes of pediatric spinal cord cavernous malformation-a report of 18 cases and literature review. *Child's Nervous System*. Jan 2021;37:235-242. doi:10.1007/s00381-020-04700-9

45. Niedermeyer S, Szelenyi A, Schichor C, Tonn JC, Siller S. Intramedullary spinal cord cavernous malformations-association between intraoperative neurophysiological monitoring changes and neurological outcome. *Acta Neurochirurgica*. Oct 2022;164(10):2595-2604. doi:10.1007/s00701-022-05354-z

46. Cai Z, Hong X, Dai W, et al. Surgical Outcomes of Symptomatic Intramedullary Spinal Cord Cavernous Malformations: Analysis of Consecutive Cases in a Single Center. *Neurospine*. Sep 2023;20:810-821. doi:10.14245/ns.2346430.215

47. Chen B, Wu C, Liu B, Yu T, Wang ZY. Prognosis of patients with spinal intramedullary cavernous hemangioma by different treatments. *Beijing da xue xue bao*. Aug 2023:652-657. doi:10.19723/j.issn.1671-167x.2023.04.014

48. Hegde A, Mohan S, Tan KK, Lim CCT. Spinal cavernous malformations: magnetic resonance imaging and associated findings. *Singap Med J*. Sep 2012;53(9):582-586.

49. Kim KM, Chung CK, Huh W, et al. Clinical outcomes of conservative management of spinal cord cavernous angiomas. *Acta Neurochirurgica*. Jul 2013;155(7):1209-1214. doi:10.1007/s00701-013-1760-7

50. Ren J, Jiang N, Bian L, et al. Natural History of Spinal Cord Cavernous Malformations: A Multicenter Cohort Study. *Neurosurgery*. Apr 2022;90:390-398. doi:10.1227/neu.0000000000001842

51. Shan Y, Ling F, Li M, Zhi X. Diagnosis and treatment of spinal cord cavernous angioma. *Zhonghua wai ke za zhi [Chinese journal of surgery]*. Apr 2002;40:287-289.

52. Labauge P, Bouly S, Parker F, et al. Outcome in 53 patients with spinal cord cavernomas. *Surgical Neurology*. Aug 2008;70:176-181. doi:10.1016/j.surneu.2007.06.039

53. Badhiwala JH, Farrokhyar F, Alhazzani W, et al. Surgical outcomes and natural history of intramedullary spinal cord cavernous malformations: a single-center series and meta-analysis of individual patient data. *J Neurosurg-Spine*. Oct 2014;21(4):662-676. doi:10.3171/2014.6.spine13949

54. Früh A, Wasilewski D, Hallek L, Wessels L, Vajkoczy P. Treatment of Spinal Cavernous Malformations: A Single-Center Case Series. *World Neurosurg*. Mar 2025;196:123758. doi:10.1016/j.wneu.2025.123758

55. Ren J, Hong T, He C, et al. Surgical approaches and long-term outcomes of intramedullary spinal cord cavernous malformations: a single-center consecutive series of 219 patients. *J Neurosurg Spine*. Apr 2019;31(1):123-132. doi:10.3171/2018.12.Spine181263
